# Comprehensive Epitope Analysis of Monoclonal Antibodies Binding to Hen Egg Ovalbumin Using a Peptide Array

**DOI:** 10.3390/foods13030407

**Published:** 2024-01-26

**Authors:** Yuko Terada, Masanobu Akimoto, Hirofumi Sakoda, Shunsuke Yamamoto, Mayuka Kubota, Tomoharu Motoyama, Yo Imanaka, Shogo Nakano, Sohei Ito, Shigeki Kato, Keisuke Ito

**Affiliations:** 1 Department of Food and Nutritional Sciences, Graduate School of Integrated Pharmaceutical and Nutritional Sciences, University of Shizuoka, 52-1 Yada, Suruga-ku, Shizuoka-shi 422-8526, Shizuoka, Japan; yukoterada@u-shizuoka-ken.ac.jp (Y.T.); kumao.6m.khmi@gmail.com (M.K.); tmotoyama1021@gmail.com (T.M.); yo.imanaka@ci-planning.co.jp (Y.I.); snakano@u-shizuoka-ken.ac.jp (S.N.); itosohei@u-shizuoka-ken.ac.jp (S.I.); 2 Research and Development Department, Prima Meat Packers, Ltd., 635 Nakamukaihara, Tsuchiura-shi 300-0841, Ibaraki, Japan; Masanobu.Akimoto@primaham.co.jp (M.A.); Hirofumi.Sakoda@primaham.co.jp (H.S.); Syunsuke.Yamamoto@primaham.co.jp (S.Y.); Shigeki.Katou@primaham.co.jp (S.K.)

**Keywords:** allergen test kit, epitope mapping, food allergy, food safety, hen egg, lateral flow immunoassay, ovalbumin, peptide array

## Abstract

Food allergies are a significant health issue worldwide. In many countries, labeling of primary allergens in food products has been made mandatory to ensure consumer safety. In food manufacturing settings, the lateral flow immunoassay (LFI)—based on antigen–antibody reactions—is a rapid and accurate method for allergen testing and is widely used. Peptide arrays are tools that enable the synthesis of peptides of any sequence on a substrate and high-throughput analysis of their interactions with chemicals. This study aimed to investigate a new application of peptide arrays in the field of food technology, particularly in the development of antibodies for food allergen testing. First, monoclonal antibodies against hen egg ovalbumin, a major food allergen, were produced. Then, using a peptide array, the epitope and specificity of the antibodies were comprehensively and precisely analyzed. Finally, an LFI kit incorporating the antibodies demonstrated both high specificity and detection sensitivity for food allergen testing. These findings indicate that peptide arrays are valuable tools in the development of antibodies for food allergen testing, ensuring reliability and accuracy at the molecular level.

## 1. Introduction

Food allergies have emerged as a significant health concern worldwide. Over the past two decades, the number of affected patients has significantly increased, with global estimates exceeding 220 million [1,2]. To ensure consumer safety, many countries have made labeling of major allergens in food products mandatory [3,4]. For instance, the European Union necessitates labeling of 14 items, including eggs, milk, and peanuts, whereas the USA and Japan mandate labeling of 8 and 7 items, respectively [5,6]. Enzyme-linked immunosorbent assay (ELISA), being recognized as a powerful tool, is considered the gold standard in many countries, including the aforementioned ones, for food allergen detection [5,7,8]. Lateral flow immunoassay (LFI) is widely employed as a simple allergen detection method [9,10]. It is extremely useful for quality control in food production sites as it enables quick and easy visualization of allergen test results without requiring expensive equipment. Both ELISA and LFI hinge on the principle of antigen–antibody reactions for allergen detection [7,11]. Epitope analysis of antibodies for food allergen detection is crucial in ensuring the reliability of the test kit at the molecular level.

Typically, epitope analyses use techniques such as peptide synthesis, followed by binding analysis to antibodies via ELISA or Western blotting [12]. However, these methodologies are notably resource-intensive and time-consuming. Although there are alternative methods such as liquid chromatography–mass spectrometry (LC-MS) for identifying the sequences of antibody-bound peptides, more rapid and convenient methodologies will likely accelerate the development of antibodies for food allergen detection. To ensure the specificity of food allergen detection antibodies, swift cross-reactivity tests are indispensable. Hence, techniques that can be used to quickly conduct epitope analysis and cross-reactivity tests for food allergen detection antibodies are warranted.

A peptide array is a tool that enables the parallel synthesis of peptides with specific sequences on substrates such as cellulose membranes and slide glasses via solid-phase synthesis [13,14]. It has a high throughput: it enables direct analysis of interactions between chemical substances and tens to hundreds of peptides on a substrate in a single measurement. De-Simone et al. synthesized a peptide library that covers the entire amino acid sequence of diphtheria toxin using the solid-phase peptide synthesis (SPOT) method. Then, they analyzed IgG binding on the substrate to perform epitope mapping of the diphtheria toxin [15]. Szymczak et al. developed a technique to rapidly analyze the susceptibility of acetylation for each peptide sequence using a peptide library synthesized employing the SPOT method [16]. Thus, while peptide arrays are becoming increasingly popular as versatile screening tools, mainly in the medical field, their potential applications are still under development. In the field of food technology, reports are scarce. However, in recent years, we have identified peptides that bind to catechin from green tea Rubisco using peptide arrays and also proposed the application of peptide arrays for bitterness masking [17].

This study investigated the novel application of peptide arrays, examining their potential use in the development of antibodies for food allergen detection. Specifically, monoclonal antibodies against hen egg ovalbumin, a major food allergen, were produced. A detailed epitope analysis was conducted via a peptide array, and cross-reactivity tests were performed. The LFI kit developed using these antibodies exhibited high sensitivity and specificity suitable for actual food allergen testing. Peptide array analysis is expected to be valuable for the development of food allergen testing antibodies whose reliability and accuracy are proven at the molecular level.

## 2. Materials and Methods

### 2.1. Reagents

The reagents used for peptide synthesis were Fmoc amino acids, N,N′-diisopropylcarbodiimide, 1-hydroxy-1H-benzotriazole, piperidine, 2,2,2-trifluoroacetic acid, and triisopropylsilane, which were purchased from Watanabe Chemical Industries, Ltd. (Hiroshima, Japan). Acetic anhydride, N,N-dimethylformamide, and N-methyl-2-pyrrolidone were obtained from FUJIFILM Wako Pure Chemical Corporation (Osaka, Japan). Dichloromethane was sourced from Kanto Chemical Co., Inc. (Tokyo, Japan).

### 2.2. Production of Anti-Hen Egg Ovalbumin Monoclonal Antibody

The anti-hen egg ovalbumin monoclonal antibody was produced according to the method described by Crestfield et al.; reduced and carboxymethylated (RCM) purified hen egg white ovalbumin was used as an antigen [18]. The RCM ovalbumin was dissolved in saline to prepare a 0.1% solution, mixed with complete Freund’s adjuvant (Sigma-Aldrich, St. Louis, MO, USA) in a 1:1 ratio, and intraperitoneally injected into female 5-week-old BALB/c mice (CLEA Japan, Inc., Tokyo, Japan). Subsequently, the solution was intraperitoneally administered to the mice every 2 weeks. Once the antibody titer had sufficiently increased, a final immunization was administered through the tail vein. Three days later, after aseptic removal of the spleen from the mouse, cell fusion was performed with mouse myeloma cells (P3-X63-Ag8.653, Riken Cell Bank, Tsukuba, Japan) according to the method described by Köhler et al. [19]. After cell fusion, the hybridomas were cultured in RPMI 1640 medium (GIBCO, Thermo Fisher Scientific, Waltham, MA, USA) supplemented with fetal bovine serum, 2-mercaptoethanol, potassium penicillin G, and streptomycin sulfate, with hypoxanthine–aminopterin–thymidine selection medium, containing hypoxanthine, aminopterin, and thymidine, at 37 °C in 5% CO_2_. Hybridomas showing a positive reaction to RCM-modified ovalbumin in a direct ELISA using culture supernatant were cloned and monoclonalized using the limiting dilution method. BALB/c mouse thymocytes were used as feeder cells. The cloned hybridomas were intraperitoneally inoculated into male 8-week-old BALB/c mice (CLEA Japan, Inc.) according to the method described by Jones et al. [20]. The obtained ascites fluid was purified using HiTrap Protein G HP (GE Healthcare, Waukesha, WI, USA), resulting in the purified monoclonal antibody. All animal experiments were conducted in accordance with the animal welfare law in Japan [21].

### 2.3. Selection of Antibody via Direct ELISA

The RCM-modified ovalbumin was adjusted to 0.1 mg/mL in phosphate-buffered saline (PBS), added to a Nunc immuno module 96-well plate (Nunc, Thermo Fisher Scientific) at 100 µL/well, and then incubated at 37 °C for 1.5 h. The wells were washed with PBS containing 0.02% Tween20 followed by blocking with 1% bovine serum albumin (BSA)/PBS. Each purified monoclonal antibody was prepared at 0.1 mg/mL, and 100 µL/well was added, followed by incubation at 37 °C for 1.5 h. After washing the wells with phosphate-buffered saline with Tween (PBST), 100 µL of secondary antibody (biotin conjugate anti-mouse IgG H+L antibody) was added, followed by incubation at 37 °C for 1.5 h. The wells were washed with PBST again, then 100 µL/well of alkaline phosphatase–streptavidin was added, and incubation was continued at 37 °C for 1.5 h. Subsequently, 50 µL/well of p-nitrophenyl phosphate disodium salt (pNPP) was added, followed by incubation at 25 °C for 30 min. After adding 100 µL/well of a mixed solution of hydrochloric and sulfuric acids, absorbance at 405 nm was measured using a plate reader (Tecan, Switzerland, Männedorf).

### 2.4. Epitope Analysis of 65F2 and 962H2 Monoclonal Antibodies Using a Peptide Array

A peptide library of arbitrary sequences was synthesized in the solid phase on a cellulose membrane using a ResPep SL peptide synthesizer (Intavis AG, Tübingen, Germany) using the Fmoc method. For example, in the epitope analysis of the 65F2 and 962H2 antibodies, peptide libraries encompassing the entire amino acid sequence of hen egg ovalbumin (UniProt: P01012) were synthesized. After immersing the peptide-synthesized cellulose membrane in MeOH, it was washed three times with Milli-Q water and once with TBS-T (20 mM Tris-HCl, pH 7.5, 150 mM NaCl, 0.05% Tween20). Then, the membrane was sequentially incubated with blocking solution (1% BSA in TBS-T), primary antibody solution (1 mg/mL 65F2, 1:3000), and secondary antibody solution (1.6 mg/mL anti-mouse IgG [Fc-specific]–alkaline phosphatase antibody produced in goat, 1:5000, Sigma-Aldrich) at 37 °C for 1 h each. After 5 min of shaking in a 5-bromo-4-chloro-3-indolyl-phosphate/nitro blue tetrazolium (BCIP/NBT) solution (Sigma-Aldrich) in the dark, the reaction was stopped by removing the solution and adding a large volume of Milli-Q water. Once dried, the staining intensity of each spot was quantified using Image J software (version 1.51h).

### 2.5. Epitope Analysis of 65F2 Monoclonal Antibody by Competitive ELISA

Using the PSSM-8 peptide synthesizer (Shimadzu, Kyoto, Japan), approximately 20 amino acid residues of peptides covering the entire amino acid sequence of hen egg ovalbumin (UniProt: P01012) and 11 peptides with 12 amino acid residues shifted by 1 residue around the epitope candidate region were synthesized using the Fmoc method. Synthesis of peptides and their cleavage from the solid phase were performed according to the manufacturer’s instructions. For the diethyl ether wash, diethyl ether was added to the obtained peptide solutions, and after centrifugation (1750 rpm, 4 °C, 5 min), the supernatant was removed; this process was repeated three times. Subsequently, the peptides were purified using Sephadex G-25 Fine (GE Healthcare, Japan, Tokyo) and then freeze-dried. 

Ovalbumin prepared at 4 µg/mL in PBS was added to a Nunc immuno module 96-well plate at 100 µL/well and incubated at 37 °C for 1.5 h. Then, the 96-well plate was washed with PBST and blocked with 1% BSA/PBS, followed by another wash with PBST. The 65F2 antibody (20 ng/mL) dissolved in PBST and ovalbumin or the peptides synthesized on the peptide array (40, 160, and 2000 µg/mL) dissolved in PBST were mixed in equal amounts and then added to the 96-well plate at 100 µL/well. Subsequently, the 96-well plate was incubated with the secondary antibody (Alkaline Phosphatase-conjugated AffiniPure Rabbit Anti-Mouse IgG (H+L), 1:5000, Sigma-Aldrich) at 100 µL/well at 37 °C for 1.5 h, followed by another wash with PBST. pNPP was added to the 96-well plate at 100 µL/well, and after incubation at 25 °C for 30 min, a 5 N sodium hydroxide solution was added at 50 µL/well. Absorbance at 405 nm was measured using a plate reader (TECAN).

### 2.6. Cross-Reactivity Test of 65F2 Antibody Using Peptides with Sequences Similar to the Epitope

Amino acid sequences of 71 major food proteins, including grains, meats, and fish, were obtained from the UniProt Knowledgebase (https://www.uniprot.org: accessed on 18 November 2016). These sequences are presented in detail in Supplementary Information S1. Among these 71 sequences, those that matched the four amino acids on the ovalbumin epitope, identified as crucial for binding with the 65F2 antibody via alanine scanning, in both arrangement and type, were screened using an in silico program. Sequences that matched the critical amino acids in arrangement and type were synthesized on the peptide array using the SPOT method and subsequently used for the cross-reactivity assay of the 65F2 antibody.

### 2.7. Production of Lateral Flow Immunoassay (LFI) Kit Using 65F2 and 962H2 Antibodies

The 65F2 antibody was utilized as the solid-phase antibody, also known as the capture antibody, in the LFI detection site, whereas 962H2 was used as the colloidal gold-labeled antibody.

Preparation of colloidal gold-labeled antibody: The 962H2 solution (500 µL, 1 mg/mL in 2 mM borate buffer, pH 9.0) was mixed with 5 mL of colloidal gold solution (Sigma-Aldrich, 0.2 M in potassium carbonate solution pH 9.0) and reacted at room temperature for 30 min. Then, 635 µL of BSA solution (10%) was added to the mixture, and the reaction was continued for an additional 15 min. After centrifugation, the supernatant was adjusted to OD525 = 1.0 using a 1% BSA solution to prepare the colloidal gold-labeled antibody solution.

LFI kit preparation: A solution of 65F2 (4 mg/mL in PBS) was linearly coated onto a nitrocellulose membrane and dried. To prepare the antibody-fixed membrane, the membrane was blocked with 0.1% BSA in TBS at 37 °C for 1 h, washed with TBS, and then dried. Separately, glass fiber sample pads for sample liquid application and glass fiber absorbent pads for sample liquid absorption were prepared. These components were then assembled in the sequence of a sample pad, antibody-anchored membrane, and absorbent pad to develop the LFI kit (Allergeneye Immunochromatography Egg for the detection of allergen in food, Prima Meat Packers, Ltd., Ibaraki, Japan).

### 2.8. Evaluation of LFI Sensitivity in Detecting Allergens in Sample Solutions

The colloidal gold-labeled 962H2 antibody solution (20 µL) was mixed with fetal bovine serum (30 µL) and freeze-dried. To prepare the test solution, the 962H2 antibody solution was mixed with 100 µL hen egg white solution (containing equivalent amounts of ovalbumin: 0, 0.5, 1, 1.5, 2.5, 4, and 5 μg/g). This test solution was applied to the LFI strip and reacted for 20 min. The test results (intensity of the judgment line) were determined visually and using an immunochromato reader (Hamamatsu Photonics K.K., Shizuoka, Japan) based on mABS values.

### 2.9. Evaluation of LFI Sensitivity in Detecting Allergens in Model Foods

Five types of processed foods (udon noodles, pork ham, processed hen breast, lactic-fermented beverage, and pumpkin soup) were prepared with the addition of hen egg white solution (containing equivalent amounts of ovalbumin: 0, 1, and 5 μg/g). Specifically, the model foods were prepared by mixing the ingredients with hen egg white solution, followed by processing operations such as vacuum packaging and heating. Detailed procedures for the preparation of each model food are provided in Supplementary Information S2. Food samples, both with and without ovalbumin (1 g each), were heated in boiling water for 10 min after adding 19 mL of allergen extraction solution (Dulbecco’s PBS containing 0.5% sodium dodecyl sulfate, 0.2% Tween20, and 0.1% sodium thiosulfate) (Prima Meat Packers, Ltd.). After cooling and centrifugation, the supernatant was subjected to the LFI kit according to the procedure presented in Section 2.8. Quantification of egg allergens in model foods was measured using Allergeneye ELISA II for Egg (Prima Meat Packers, Ltd.) [7].

### 2.10. Cross-Reactivity of the LFI Kit

The cross-reactivity of the LFI kit was assessed across 72 types of foods containing major food allergens, e.g., milk, peanut, and wheat. Each food sample (1 g) used in the cross-reactivity test was heated in boiling water for 10 min after the addition of 19 mL of an allergen extraction solution (Prima Meat Packers, Ltd.), which consisted of 0.5% sodium dodecyl sulfate, 0.2% Tween20, and 0.1% sodium thiosulfate in Dulbecco’s PBS. After cooling and centrifugation, the supernatant was subjected to the LFI kit according to the procedure presented in Section 2.8.

### 2.11. Validation of the Practicality of the LFI Kit Using Commercially Available Food Products

To validate the practicality of the LFI kit developed in this study, analyses were conducted using 20 different commercially available food products. Ten products labeled as containing egg (bread, instant noodles, pasta sauce, pudding, potato salad, Chinese dumplings, fried rice, egg soup, sandwich, whipped cream mochi) and ten egg-free products (bread, instant noodles, pasta sauce, fruit jelly, vegetable salad, rice ball, potato chips, gratin, fried snack, strawberry jam) were used. Each food product was homogenized using a food processor, and 1 g of the homogenate was prepared for analysis following the procedure described in Section 2.9 and then provided to the LFI kit. The LFI test results (intensity of the judgment line) were determined visually. Hen egg protein in each food product was quantified by the ELISA method as described in Section 2.9.

## 3. Results and Discussion

### 3.1. Selection of Monoclonal Antibodies against Hen Egg Ovalbumin Using Direct ELISA

**Figure 1:**
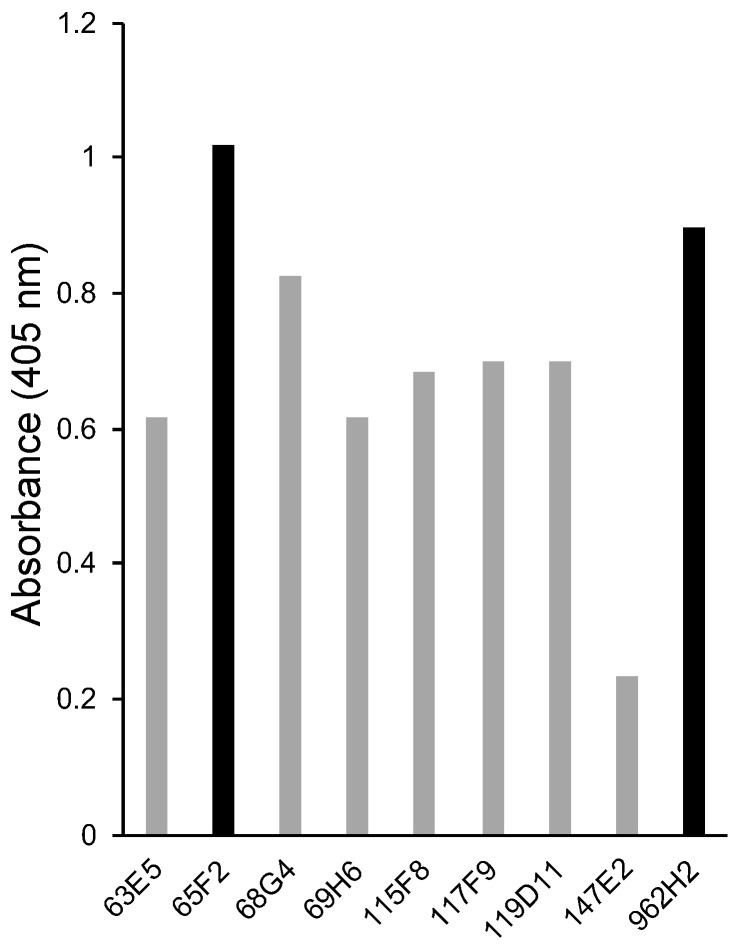
Selection of anti-ovalbumin monoclonal antibodies via direct ELISA. The reactivity of the nine prepared monoclonal antibodies against hen egg white ovalbumin was analyzed via direct ELISA. The vertical axis represents absorbance at 405 nm.

For the development of a highly sensitive LFI for hen egg ovalbumin, monoclonal antibodies against hen egg ovalbumin were initially selected. The reactivity of the nine monoclonal antibodies against hen egg ovalbumin obtained in Section 2.2 was analyzed via ELISA. Among them, 65F2 exhibited the highest reactivity, followed by 962H2 (Figure 1). Based on these observations, 65F2 and 962H2 were selected as model antibodies for a detailed epitope analysis via a peptide array. When sandwich ELISA was performed using a combination of the two antibodies, 65F2 and 962H2, high reactivity against hen egg ovalbumin was observed. Therefore, 65F2 and 962H2 were used as solid-phase and colloidal gold-conjugated antibodies, respectively, for an LFI kit to detect hen eggs. In the ELISA analysis, several monoclonal antibodies, including 68G4, showed moderate reactivity. However, due to the high reactivity observed in sandwich ELISA using the combination of 65F2 and 962H2, no further analysis was performed on antibodies with lower reactivity.

### 3.2. Epitope Analysis of Monoclonal Antibodies against Hen Egg Ovalbumin Using a Peptide Array

**Figure 2:**
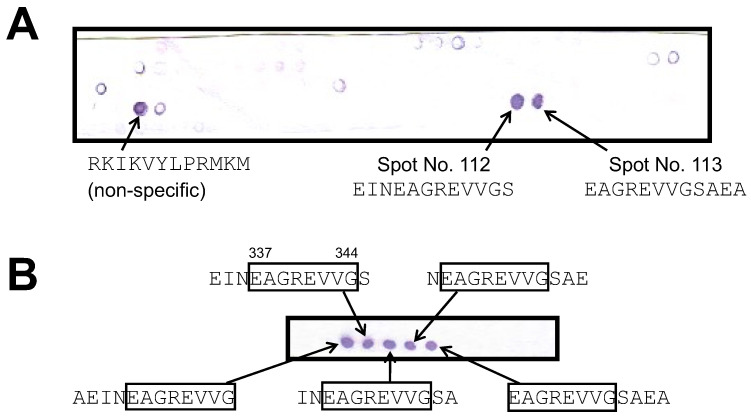
Comprehensive analysis of the epitope on ovalbumin for the 65F2 antibody using a peptide array. (**A**) First phase of epitope analysis. Peptides consisting of twelve amino acids with a three-amino-acid shift, covering the entire amino acid sequence of hen egg ovalbumin, were SPOT-synthesized on a cellulose membrane. Binding of the 65F2 antibody was detected via BCIP/NB staining. (**B**) Second phase of epitope analysis. Sequences around spot Nos. 112 and 113, which were identified as the epitope candidate regions in the first phase, were synthesized as peptides of 12 amino acids with a 1-amino-acid shift. The binding of the 65F2 antibody was detected via BCIP/NB staining. The amino acid sequence common to the positive spots, the epitope candidate region, is encircled.

Using 65F2, a monoclonal antibody against hen egg ovalbumin, as a model, the utility of a peptide array in epitope analysis for food allergen-binding antibodies was explored. First, using a peptide array, a total of 126 peptides, each comprising twelve amino acids and offset by three amino acids, were synthesized to cover the entire sequence of hen egg ovalbumin on a cellulose membrane using the SPOT method. Then, these 126 peptides were reacted with the 65F2 antibody. After the reaction, the alkaline phosphatase-labeled secondary antibody was reacted with them, followed by BCIP/NB staining. As a result of the analysis, positive reactions were detected for spot Nos. 112 and 113 (Figure 2A). Notably, the spot for “RKIKVYLPRMKM” was detected even with the secondary antibody alone; thus, it was classified as nonspecific. To determine the candidate epitope region, 16 peptides, each comprising 12 amino acids and shifted by one residue, were synthesized around the sequences of spot Nos. 112 and 113 using the SPOT method. These peptides were then analyzed similarly. The five positive spots detected consistently contained “EAGREVVG” (positions 337–344) (Figure 2B). The results indicated that “EAGREVVG” (positions 337–344) is the potential epitope region for the 65F2 antibody. Using a similar methodology, the candidate epitope region for 962H2, the second most sensitive antibody against hen egg ovalbumin after 65F2, was examined. “VLQPSS” (positions 161–166) was identified as the potential epitope region for 962H2 (Supplementary Figure S1).

### 3.3. Precise Epitope Analysis via Deletion Analysis

**Figure 3:**
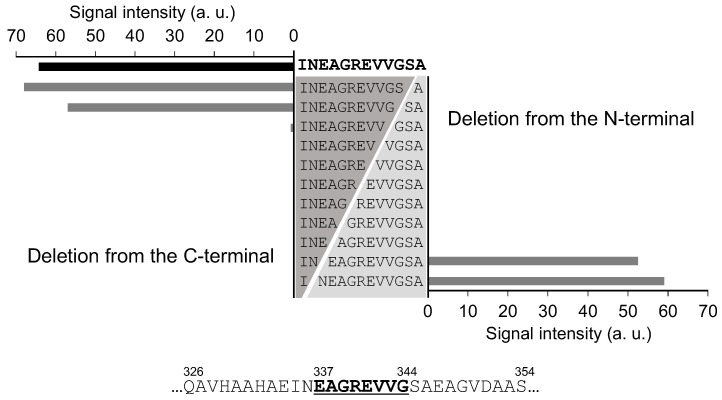
Precise analysis of the 65F2 antibody epitope via deletion analysis. To identify the minimum amino acid region in the epitope that contributes to binding with the 65F2 antibody, a deletion analysis was conducted. After SPOT synthesis of peptides on a cellulose membrane with one amino acid removed from both the N- and C-termini of the sequence “INEAGREVVGSA”, the binding of the 65F2 antibody was detected via BCIP/NB staining.

To identify the minimal amino acid region within the epitope that contributes to binding with the 65F2 and 962H2 antibodies, peptides were synthesized on a peptide array, each sequentially truncated by one amino acid from either the N- or C-terminus of the 65F2 candidate epitope region “INEAGREVVGSA” (positions 335–346). Binding activity to the 65F2 antibody was nearly lost due to the absence of E337 and G344, which led to the identification of “EAGREVVG” (positions 337–344) as the precise epitope (Figure 3). A similar deletion analysis was conducted for the potential epitope region of the 962H2 antibody, “IRNVLQPSSVDS” (positions 158–169). The loss of binding capability to 962H2 owing to the deletion of V161 and D168 indicated that “VLQPSSVD” (positions 161–168) is the candidate epitope region for 962H2 (Supplementary Figure S2).

The Immune Epitope Database (IEDB) lists 171 human-derived and 1,511 mouse-derived sequences as epitopes against hen egg ovalbumin (Gal d 2) from human and mouse antibodies, respectively (https://www.iedb.org: accessed on 22 December 2023). The sequence “EAGREVVG” was identified as a candidate epitope region for the 95F2 antibody and is recognized as an epitope for hen egg ovalbumin from mouse-derived antibodies, as registered in the IEDB [22]. The aforementioned sequence is also included in the epitope “VHAAHAEINEAGREVVGS” for ovalbumin, recognized by IgE derived from patients with a hen egg allergy [23]. Regarding the candidate epitope region “VLQPSSVD” for the 962H2 antibody, the sequence itself was not listed in the IEDB. This sequence was included in two epitopes against hen egg ovalbumin derived from mice, as reported by Clement [22]. Furthermore, among the IgE epitopes from patients with hen egg allergy reported by Suprun et al., “VLQPSSVD” appeared in 64 sequences [23]. Consequently, the epitope regions identified for the 65F2 and 962H2 antibodies are sequences common in epitopes derived from both human and mouse antibodies.

### 3.4. Epitope Analysis by Competitive ELISA and Validation of Peptide Array Analysis Method

Generally, the competitive ELISA method is used for the epitope analysis of antibodies for food allergen tests [5,7,24]. To validate the results of epitope analysis using the peptide array, a comparison was made with the results obtained using the competitive ELISA method. Initially, 18 types of epitope candidate peptides, each consisting of 20 amino acids covering the entire amino acid sequence of hen egg white ovalbumin, were synthesized and subjected to competitive ELISA. One of the peptides exhibited competitive activity against 65F2. Subsequently, for the sequence that showed competitive activity, 11 peptides shifted by one amino acid were synthesized and subjected to competitive ELISA. The analysis revealed that because the competitive activity was lost due to the absence of N336 and G344, “NEAGREVVG” (positions 336–344) was classified as the epitope for 65F2 (Supplementary Figure S3). This sequence was almost identical to the epitope “EAGREVVG” (positions 337–344) identified using the peptide array (Table 1). These results suggest that the method using a peptide array for the epitope analysis of antibodies for food allergen tests has an accuracy equivalent to the competitive ELISA method. Although competitive ELISA is a common method for epitope analysis [5,7,24], comprehensive analysis of epitopes across the entire sequence of the target protein for each antibody is impractical due to the costs and effort. In addition, competitive ELISA indirectly detects the binding of antibodies and peptides, and direct evidence of their binding is not obtained. Conversely, the peptide array, which synthesizes various peptides in parallel on a substrate, can be described as a method superior in “comprehensiveness, speed, and directness.”

### 3.5. Analysis of Epitope-Constituting Amino Acids Critical for Antibody Binding by Alanine Scanning

**Figure 4:**
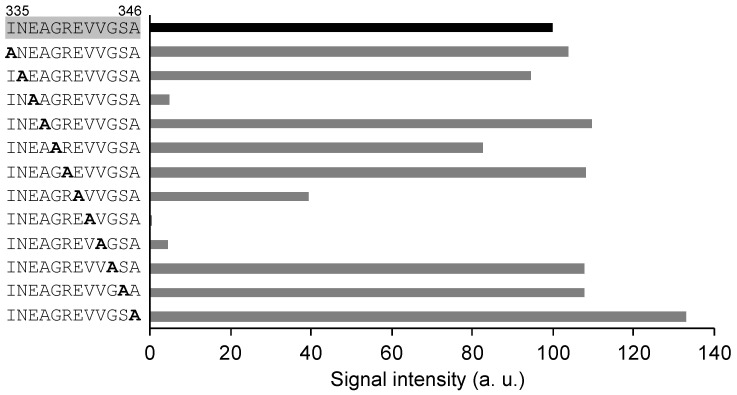
Analysis of amino acids critical for the binding of the 65F2 antibody via alanine scanning. To identify the amino acids crucial for the 65F2 antibody binding, each amino acid in the epitope region was replaced by alanine in separate peptides. Binding of the 65F2 antibody to the unaltered sequence “INEAGREVVGSA” was set as 100 (black bar), and the antibody binding to each alanine-replaced peptide is shown as a gray bar, represented as a relative value.

To analyze the contribution of specific epitope-constituting amino acids to antibody binding, alanine scanning was performed using a peptide array. The sequence surrounding the 65F2 epitope “INEAGREVVGSA” (positions 335–346) was subjected to alanine scanning. The substitution of E337, E341, V342, and V343 by alanine reduced the binding of the 65F2 antibody by 95%, 60%, 99.5%, and 95%, respectively (Figure 4), suggesting that these four amino acids are crucial for 65F2 binding. The region surrounding the epitope “IRNVLQPSSVDS” (positions 158–169) for 962H2 was analyzed similarly. The substitution of alanine for I158, V161, L162, Q163, P164, V167, and D168 resulted in a greater reduction in antibody binding strength compared to when other amino acid residues were replaced with alanine (Supplementary Figure S4). Although I158 is outside the epitope region, the reduction in 962H2 binding owing to its substitution with alanine indicates that the area surrounding the epitope is also involved in 962H2 binding. In particular, the alanine substitution at V161, L162, Q163, and P164 significantly decreased the binding intensity of the antibody, suggesting that these four amino acids are crucial for 962H2 binding.

Mine et al. conducted a detailed analysis of epitopes in hen egg ovalbumin for human and mouse IgE antibodies. They reported that among the amino acids constituting the epitope, the occurrence frequency of hydrophobic amino acids is the highest, about 55%. Furthermore, they found that hydrophobic amino acids, particularly A, V, and F, played a significant role in antibody binding [25,26]. In our alanine scanning, the amino acids determined as critically important for binding with the 65F2 and 962H2 antibodies were 65F2 (E337, E341, V342, and V343) and 962H2 (V161, L162, Q163, and P164), respectively. The proportion of hydrophobic amino acids was 2/4 (50%) for 65F2 and 3/4 (75%) for 962H2. Based on the report by Mine et al., it can be inferred that these hydrophobic amino acids are involved in the formation of hydrophobic interactions between the antibody and the epitope.

A detailed analysis of the binding mechanism between antibodies and epitopes is necessary for the development of food allergen test antibodies with high accuracy at the molecular level. Analysis using a peptide array enabled a rapid and straightforward identification of regions centrally contributing to antibody binding at the single-amino acid level.

### 3.6. Cross-Reactivity Test of 65F2 Antibody Using Peptide Array

To analyze the specificity of the 65F2 antibody, its binding to epitope-like sequence peptides was examined. Initially, amino acid sequence information for 71 types of major food proteins, such as grains and meats, was obtained from the UniProt Knowledgebase. Among these 71 sequences, those matching the four amino acids (E337, E341, V342, and V343)—identified as crucial for binding with the 65F2 antibody through alanine scanning in Section 3.5—in terms of both amino acid arrangement and type were screened using an in silico program. 

**Figure 5:**
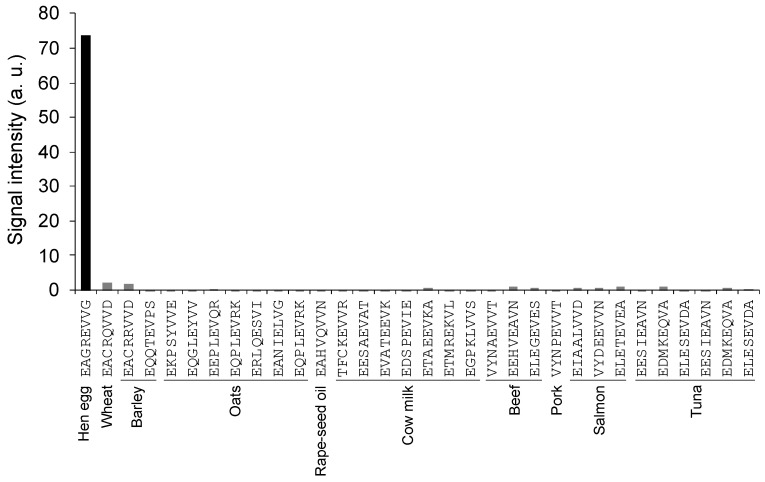
Cross-reactivity test of the 65F2 antibody using a peptide array. Among the 71 major food proteins analyzed, 31 exhibited similarity to the 65F2 epitope sequence based on in silico analysis. After SPOT synthesizing these 31 peptides, binding of the 65F2 antibody to each peptide was detected.

While no sequences matched all four amino acids necessary for binding with 65F2, 31 sequences shared both the same arrangement and type of three amino acids. The binding between these peptides and the 65F2 antibody was then analyzed using a peptide array. As 65F2 did not bind to these 31 peptides (Figure 5), it was inferred that 65F2 has high specificity for “EAGREVVG” derived from hen egg white ovalbumin.

### 3.7. Lateral Flow Immunoassay Using the 65F2 and 962H2 Antibodies

Analysis using the peptide array revealed that the 65F2 and 962H2 antibodies have a precisely defined epitope. Subsequently, an LFI kit for food allergen testing using the 65F2 and 962H2 antibodies was developed, and its sensitivity and cross-reactivity were tested. In sample solutions containing hen egg white ovalbumin (0–5 μg/g), positive detection was observed at 0.5 μg/g, and clear visual positive detection was possible at 1 μg/g or more (Table 2). The five model foods with added hen egg white ovalbumin (0, 1, and 5 μg/g) all exhibited a positive result at 1 μg/g or more (Table 3), suggesting the ability of the kit to detect allergens in foods.

The detection sensitivity of LFI kits for food allergen testing, namely, Rapid Test Pro II for Egg (Morinaga Institute of Biological Science, Inc., Yokohama, Japan) and FASTKIT Slim Egg (NH Foods Ltd., Osaka, Japan), was 5 µg/g of egg protein, which was equivalent to 2.5 µg/g of ovalbumin [27,28,29]. The LFI kit developed in this study exhibited superior or equivalent detection sensitivity, which was 1 μg/g of ovalbumin or more. In Japan, the Cabinet Office has stipulated that the amount of allergenic proteins in food is considered positive if it is 10 µg/g or 10 µg/mL or more [5,7]. Therefore, the developed LFI kit had a practically applicable detection sensitivity.

In the next step, the cross-reactivity of the developed LFI kit was tested. Upon analysis of cross-reactivity to 72 major foods containing food allergens aside from hen eggs, the LFI kit only exhibited a positive reaction to hen eggs (Table S1). It did not show cross-reactivity to other foods, suggesting that the developed LFI kit has high specificity to hen eggs.

Finally, the practical applicability of the developed LFI kit was validated using 20 commercially available food products. Ten products labeled as containing egg and ten egg-free products were tested. The results showed that all products labeled as containing egg tested positive, while the egg-free products tested negative, demonstrating that the LFI kit’s readings were consistent with the product labels (Table 4). Subsequently, the hen egg protein content in these products was quantified using the ELISA method. In the egg-free products, the egg protein content was less than 1.0 µg/g, aligning with their labels. Among the products labeled as containing egg, nine, excluding whipped cream mochi, contained high levels of egg protein, over 20 µg/g. The egg protein content in whipped cream mochi was 7.4 µg/g, which was lower than that in the other samples. Its label indicated that eggs were included as only a part of its ingredients, which likely accounts for the relatively lower egg protein content. The tests with commercial food products showed that the LFI kit results are consistent with both the food labeling and ELISA outcomes, demonstrating its applicability for detecting a range of egg protein concentrations, from low to high. Thus, the LFI kit was shown to be practically viable.

The epitope of the 65F2 and 962H2 antibodies was exhaustively and precisely analyzed using a peptide array. The 65F2 antibody exhibited high specificity in the allergen peptide cross-reactivity test based on its epitope information. Furthermore, the LFI kit using the antibodies showed practical levels of specificity and sensitivity for food allergen testing. These results indicate that comprehensive and precise epitope analysis using a peptide array and the subsequent cross-reactivity analysis based on it are a valuable tool that expedites the development of food allergen test antibodies with high accuracy.

Peptide arrays might not be applicable for conformational epitopes [30]. Furthermore, when using peptide arrays, the antibody binding test is conducted with the peptide’s C-terminus bound to the substrate [30]. This means that if the C-terminus of the protein is the epitope, the antibody might not recognize it. If candidate epitopes are not identified through peptide array analysis, it is advisable to consider the potential for C-terminal or conformational epitopes and proceed with epitope characterization using ELISA. Despite these limitations, the exhaustiveness and speed of peptide array analysis are thought to effectively compensate for the drawbacks of traditional methods, such as competitive ELISA, Western blot, and LC-MS analysis, which suffer from low throughput. Given these factors, it is anticipated that integrating peptide array analysis with traditional methods can accelerate the development of food allergen detection antibodies with reliability at the molecular level.

## Figures and Tables

**Table 1:** Epitope of the 65F2 antibody identified by peptide array and competitive ELISA. The epitope of the 65F2 antibody on hen egg ovalbumin was identified and compared using a peptide array and competitive ELISA.

Method	65F2
Peptide array	EAGREVVG
Competitive ELISA	NEAGREVVG

**Table 2:** Sensitivity of the LFI kit using the 65F2 antibody in the test solutions. The sensitivity of the LFI kit was determined via visual observation and using an immunochromatography reader (mABS) for the test solutions containing 0, 0.5, 1, 1.5, 2.5, 4, and 5 µg/g of hen egg ovalbumin. The results of visual judgment are presented as negative (−) and positive (+).

Detection Method	Ovalbumin Concentration (µg/g)						
	5	4	2.5	1.5	1	0.5	0
	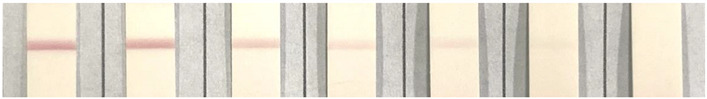						
Visual judgment	+	+	+	+	+	+	−
mABS	165.2	136.8	98.7	52.5	31.3	12.1	0

**Table 3:** Detection sensitivity of the LFI kit in the model foods. The sensitivity of the LFI kit was determined visually and using an immunochromatography reader (mABS) for five model foods containing 0, 1, and 5 µg/g of hen egg ovalbumin. The content of hen egg protein in the samples was quantified via quantitative ELISA. The results of visual judgment are presented as negative (−) and positive (+).

Model Foods	Added Ovalbumin Concentration (µg/g)	Immunochromatography Visual Judgment	Immunochromatography mABS	Quantitative ELISA Hen Egg Protein Concentration (µg/g)
Udon noodles	0	−	0	0
	1	+	33.4	2.3 (equivalent to 1.15 µg/g ovalbumin)
	5	+	143.8	9.0 (equivalent to 4.5 µg/g ovalbumin)
Pork ham	0	−	0	0
	1	+	28.4	1.9 (equivalent to 0.95 µg/g ovalbumin)
	5	+	129.9	8.5 (equivalent to 4.25 µg/g ovalbumin)
Processed chicken breast	0	−	0	0
	1	+	24.6	1.8 (equivalent to 0.9 µg/g ovalbumin)
	5	+	118.2	9.2 (equivalent to 4.6 µg/g ovalbumin)
Lactic-fermented beverage	0	−	0	0
	1	+	29.1	2.2 (equivalent to 1.1 µg/g ovalbumin)
	5	+	132.7	8.7 (equivalent to 4.35 µg/g ovalbumin)
Pumpkin soup	0	−	0	0
	1	+	31.9	2.6 (equivalent to 1.3 µg/g ovalbumin)
	5	+	140.5	9.4 (equivalent to 4.7 µg/g ovalbumin)

**Table 4:** Consistency of the results of the LFI kit with allergen labeling and ELISA in commercially available food products. The practical applicability of the LFI kit was validated using 10 products labeled as containing egg and 10 egg-free products. The results of the LFI visual judgment are presented as negative (−) or positive (+).

Commercially Available Food Products	Allergen Labeling	Immunochromatography Visual Judgment	Quantitative ELISA Hen Egg Protein Concentration (µg/g)
Bread	Egg	+	>20
Instant noodles	Egg	+	>20
Pasta sauce	Egg	+	>20
Pudding	Egg	+	>20
Potato salad	Egg	+	>20
Chinese dumplings	Egg	+	>20
Fried rice	Egg	+	>20
Egg soup	Egg	+	>20
Sandwich	Egg	+	>20
Whipped cream mochi	Egg (partial ingredient)	+	7.4
Bread	Egg-free	−	<1.0
Instant noodles	Egg-free	−	<1.0
Pasta sauce	Egg-free	−	<1.0
Fruit jelly	Egg-free	−	<1.0
Vegetable salad	Egg-free	−	<1.0
Rice ball	Egg-free	−	<1.0
Potato chips	Egg-free	−	<1.0
Gratin	Egg-free	−	<1.0
Fried snack	Egg-free	−	<1.0
Strawberry jam	Egg-free	−	<1.0

## Data Availability

The data underlying this article are available in the article and in its online Supplementary Materials.

## Supplementary Materials

The following supporting information can be downloaded at https://www.mdpi.com/article/10.3390/foods13030407/s1, Figure S1: Epitope analysis for the 962H2 antibody on ovalbumin using a peptide array; Figure S2: Detailed analysis of the 962H2 antibody epitope using deletion analysis; Figure S3: Epitope analysis of the 65F2 antibody against ovalbumin using competitive ELISA; Figure S4: Examination of amino acids essential for 962H2 antibody binding using alanine scanning; Supplementary Table S1: Cross-reactivity test of the LFI kit against major allergen foods; Supplementary Information S1: Amino acid sequences of 71 major food proteins; Supplementary Information S2: Model food preparation methods.
