# Supplementary material for: Comprehensive Epitope Analysis of Monoclonal Antibodies Binding to Hen Egg Ovalbumin Using a Peptide Array
Source: Foods. 2024 Jan 26;13(3):407. doi: 10.3390/foods13030407 (PMC10855139; doi:10.3390/foods13030407)
Supplement: Supplementary file 1 [file foods-13-00407-s001.zip › 240126_Suppl_Table 1.pdf]

Supplementary Table S1. Cross-reactivity test of the LFI kit against major allergen foods. The cross-reactivity of the LFI kit was analyzed for 72 major allergen foods, including eggs, dairy products, seafood, and meats. + indicates positive, and - indicates negative.

Category	Foods	Judgment
Eggs	Chicken egg	+
Meats	Chicken	-
	Pork	-
	Pork liver	-
	Beef	-
Dairy products	Milk	-
	Butter	-
Seafoods	Tuna	-
	Salmon	-
	Shishamo smelt	-
	Dried sardine	-
	Codfish	-
	Seabream	-
	Bonito	-
	Black tiger prawn	-
	Salted salmon caviar	-
	Salmon caviar	-
	Octopus	-
	Spear squid	-
Seaweeds	Raw brown seaweed	-
	Raw laver	-
Beans	Red beans (Adzuki)	-
	Black soybean	-
	Kidney Bean	-

Category	Foods	Judgment
Seeds	Peanut	-
	Almond	-
	Cashews	-
	Pistachio	-
	Pine nut	-
	Pumpkin seeds	-
	Sesame	-
Grains	Non-glutinous rice	-
	Buckwheat	-
	Common millet	-
	Japanese barnyard millet	-
	Foxtail millet	-
	Quinoa	-
	Wheat	-
	Rye	-
	Barley	-
	Oats	-
	Adlay	-
	Soybean	-
Spice	Cayenne pepper powder	-
	White pepper	-
	Cumin	-
	Coriander	-
	Cardamom	-

Category	Foods	Judgment
Fruits	Orange	-
	Kiwifruit	-
	Gold kiwi	-
	Avocado	-
	Banana	-
	Apple	-
	Grapefruit	-
	Mango	-
Vegetable	Spinach	-
	Onion	-
	Tomato	-
	Chinese radish	-
	Carrot	-
	Pumpkin	-
	Green onion	-
	Chinese cabbage	-
	Lotus root	-
	Sweet potato	-
	Garland chrysanthemum	-
	Burdock root	-
	Potato	-
	Shiitake mushroom	-
	Enoki mushroom	-

Cowpea	-
