# Supplementary material for: Comprehensive Epitope Analysis of Monoclonal Antibodies Binding to Hen Egg Ovalbumin Using a Peptide Array
Source: Foods. 2024 Jan 26;13(3):407. doi: 10.3390/foods13030407 (PMC10855139; doi:10.3390/foods13030407)
Supplement: Supplementary file 1 [file foods-13-00407-s001.zip › Suppl Figure_PDF.pdf]

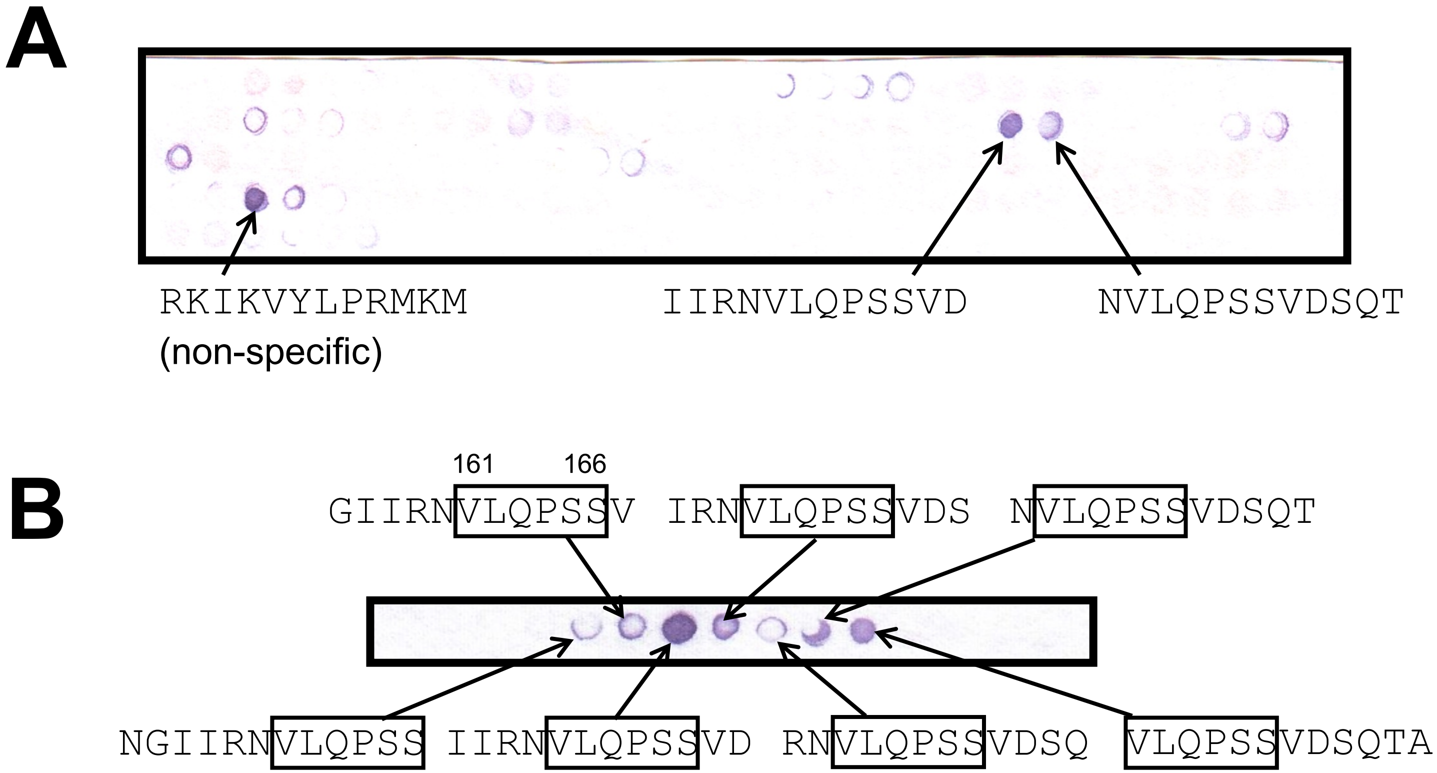

Supplementary Figure S1. Epitope analysis for the 962H2 antibody on ovalbumin using a peptide array. (**A**) First phase of epitope analysis. Peptides, each comprising 12 amino acids and offset by three residues, spanning the full amino acid sequence of hen egg ovalbumin, were synthesized on a cellulose membrane. The binding of the 962H2 antibody was visualized via BCIP/NB staining. (**B**) Second phase of epitope analysis. Peptides based on the sequences surrounding "IIRNVLQPSSVD" and "NVLQPSSVDSQT," which were identified as potential epitope regions in the first-phase analysis, were synthesized. These peptides comprised 12 amino acids and shifted by one residue. Interaction with the 962H2 antibody was again assessed via BCIP/NB staining. The shared amino acid sequence in the positively stained spots, indicating the probable epitope region, was highlighted.

Supplementary Figure S1

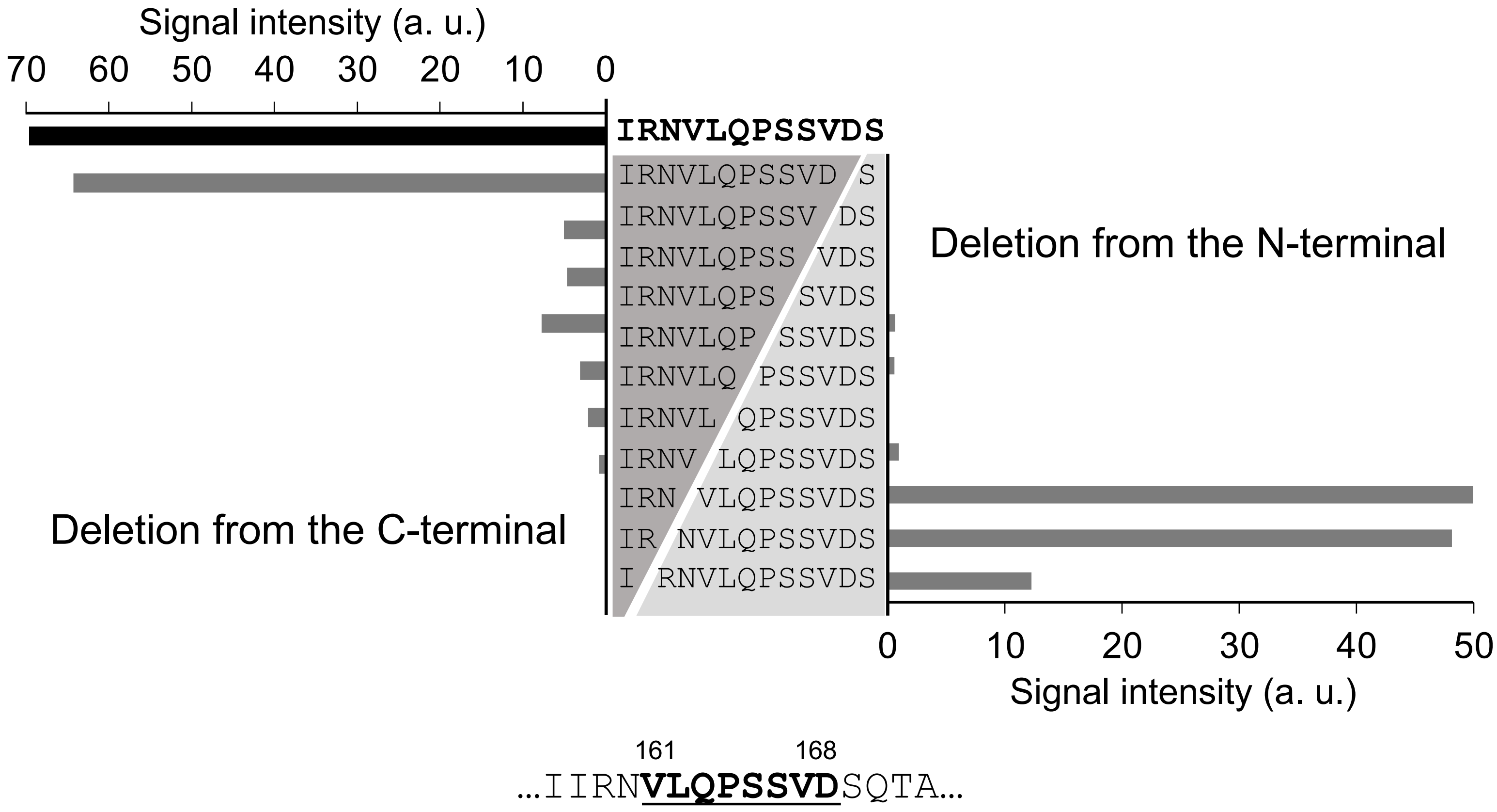

Supplementary Figure S2. Detailed analysis of the 962H2 antibody epitope using deletion analysis. To determine the smallest amino acid region within the epitope crucial for 962H2 antibody binding, peptides with one amino acid removed from both the N- and C-termini of the sequence "IRNVLQPSSVDS" were SPOT-synthesized on a cellulose membrane. The binding of the 962H2 antibody was subsequently detected via BCIP/NB staining.

Supplementary Figure S2

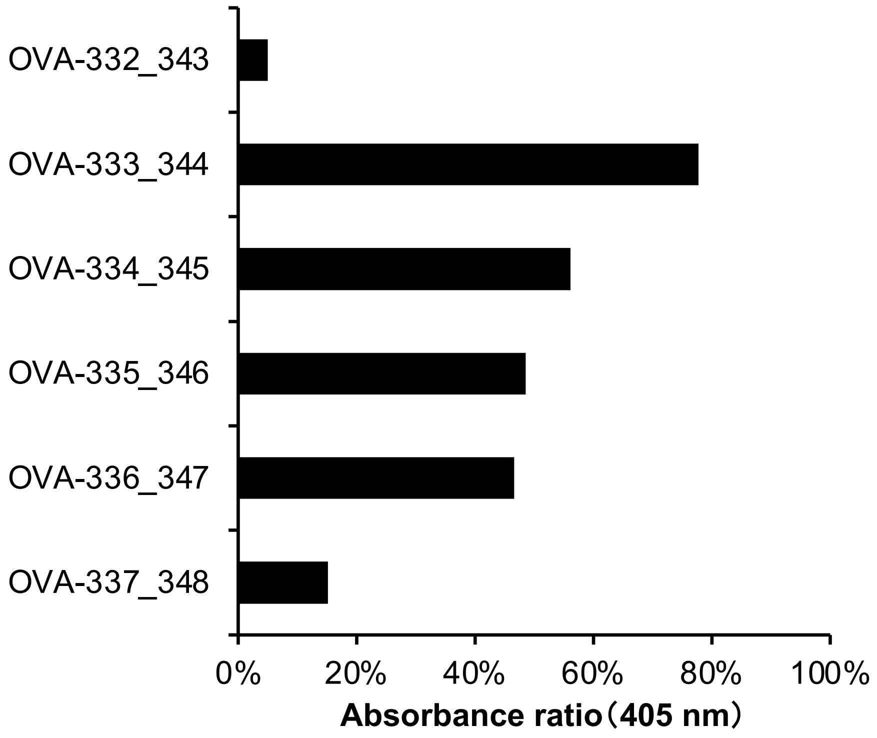

326 336 344 354

...QAVHAAHAEI**NEAGREVVG**SAEAGVDAAS...

Supplementary Figure S3. Epitope analysis of the 65F2 antibody against ovalbumin using competitive ELISA. After the initial epitope analysis, which revealed the epitope candidate region via competitive ELISA, 11 peptides, each comprising 12 amino acids and shifted by one amino acid around the identified regions, were synthesized on a peptide array. These peptides were then subjected to competitive ELISA to further investigate the epitope binding of the 65F2 antibody.

Supplementary Figure S3

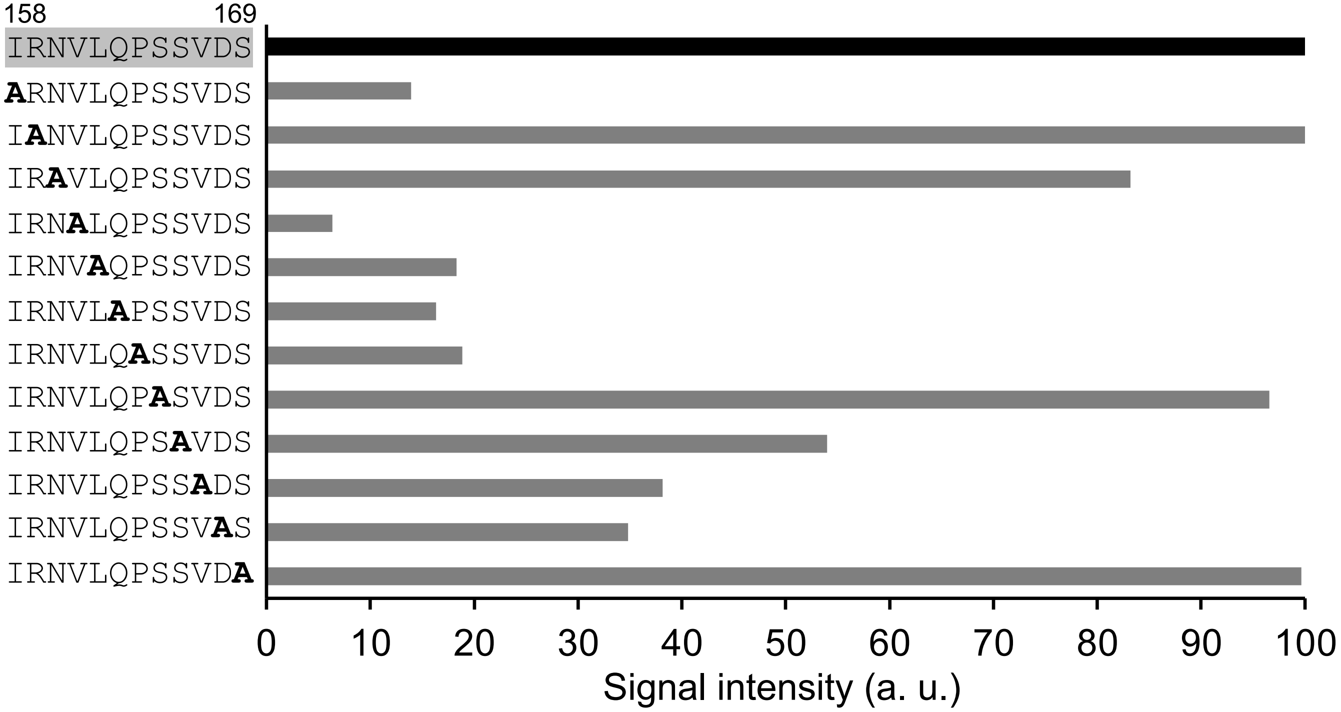

Supplementary Figure S4. Examination of amino acids essential for 962H2 antibody binding using alanine scanning. To determine which amino acids are necessary for the binding of the 962H2 antibody, each residue in the epitope region was replaced with alanine in individual peptides. The binding affinity of the 962H2 antibody to the original sequence "IRNVLQPSSVDS" was set as 100% (black bar). The binding to each alanine-substituted peptide is shown as a gray bar, indicating relative binding intensity.

Supplementary Figure S4
