# Supplementary material for: Comprehensive Epitope Analysis of Monoclonal Antibodies Binding to Hen Egg Ovalbumin Using a Peptide Array
Source: Foods. 2024 Jan 26;13(3):407. doi: 10.3390/foods13030407 (PMC10855139; doi:10.3390/foods13030407)
Supplement: Supplementary file 1 [file foods-13-00407-s001.zip › Supplementary information 1.pdf]

**Supplementary information 1.** Amino acid sequences of 71 major food proteins. Amino acid sequences of 71 major food proteins, including grains, meats, and seafood, were obtained from the UniProt Knowledgebase (https://www.uniprot.org). These 71 sequences were subjected to in silico analysis, and those with sequences similar to the epitope of the 65F2 antibody on hen's egg ovalbumin were analyzed.

Wheat_α/β- Gliadin (uniprot: P02863)
MKTFLILVLLAIVATTATTAVRFPVPQLQPQNPSQQQPQEQVPLVQQQQFLGQQQPFPPQ
QPYPQPQPFPSQLPYLQLQPFPQPQLPYSQPQPFRPQQPYPQPQPQYSQPQQPISQQQQQ
QQQQQQQQQQQQQILQQILQQQLIPCMDVVLQQHNIAHGRSQVLQQSTYQLLQELCC
QHLWQIPEQSQCQAIHNVVHAIILHQQQKQQQQPSSQVSFQQPLQQYPLGQGSFRPSQQ
NPQAQGSVQPQQLPQFEEIRNLALQTLPAMCNVYIPPYCTIAPFGIFGTN

Wheat_Glutenin, high molecular weight subunit 12 (uniprot: P08488)
MAKRLVLFAAVVIALVALTTAEGEASRQLQCERELQESSLEACRQVVDQQLAGRLPWS
TGLQMRCCQQLRDVSAKCRSVAVSQVARQYEQTVVPPKGGSFYPGETTPLQQLQQGIF
WGTSSQTVQGYYPSVTSPRQGSYYPGQASPQQPGQGQQPGKWQEPGQGQQWYYPTSL
QQPGQGQQIGKGKQGYYPTSLQQPGQGQQIGQGQQGYYPTSPQHTGQRQQPVQGQQI
GQGQQPEQGQQPGQWQQGYYPTSPQQLGQGQQPGQWQQSGQGQQGHYPTSLQQPG
QGQQGHYLASQQQPAQGQQGHYPASQQQPGQGQQGHYPASQQQPGQGQQGHYPAS
QQEPGQGQQGQIPASQQQPGQGQQGHYPASLQQPGQQGHYPTSLQQLGQGQQIGQPG
QKQQPGQGQQTGQGQQPEQEQQPGQGQQGYYPTSLQQPGQGQQQGQGQQGYYPTSL
QQPGQGQQGHYPASLQQPGQGQGQPGQRQQPGQGQHPEQGQQPGQGQQGYYPTSPQ
QPGQGQQLGQGQQGYYPTSPQQPGQGQQPGQGQQGHCPMSPQQTGQAQQLGQGQQI
GQVQQPGQGQQGYYPTSLQQPGQGQQSGQGQQSGQGHQPGQGQQSGQEKQGYDSPY
HVSAEQQAASPMVAKAQQPATQLPTVCRMEGGDALSASQ

Wheat_Glutenin, low molecular weight subunit 1D1 (uniprot: P10386)
MKTFLVFALLAVAATSAIAQMETRCIPGLERPWQQQPLPPQQTFPQQPLFSQQQQQLFP
QQPSFSQQQPPFWQQQPPFSQQQPILPQQPPFSQQQQLVLPQQPPFSQQQQPVLPPQQSP
FPQQQQQHQQLVQQQIPVVQPSILQQLNPCKVFLQQQCSPVAMPQRLARSQMLQQSSC
HVMQQQCCQQLPQIPQQSRYEAIRAIIYSIILQEQQQVQGSIQSQQQQPQQLGQCVSQPQ
QQSQQQLGQQPQQQQLAQGTFLQPHQIAQLEVMTSIALRILPTMCSVNVPLYRTTTSVP

FGVGTGVGAY

Wheat_Large subunit RuBisCO (uniprot: P11383)

MSPQTETKAGVGFKAGVKDYKLTYYTPEYETKDTDILAAFRVSPQPGVPPEEAGAAVA
AESSTGTWTTVWTDGLTSLDRYKGRCYHIEPVAGEDSQWICYVAYPLDLFEEGSVTNM
FTSIVGNVFGFKALRALRLEDLRIPPTYSKTFQGPPHGIQVERDKLNKYGRPLLGCTIKP
KLGLSAKNYGRACYECLRGGLDFTKDDENVNSQPFMRWRDRFVFCAEAIYKSQAETG
EIKGHYLNATAGTCEEMIKRAVFARELGVPIVMHDYLTGGFTANTTLAHYCRDNGLLLH
IHRAMHAVIDRQKNHGMHFRVLAKALRMSGGDHIHSGTVVGKLEGEREMTLGFVDLL
RDDFIEKDRARGIFFTQDWVSMPGVIPVASGGIHVWHMPALTEIFGDDSVLQFGGGTLG
HPWGNAPGAAANRVALEACVQARNEGRDLAREGNEIIRAACKWSPELAAACEVWKAI
KFEFEPVDTIDK

Wheat_Small subunit RuBisCO (uniprot: P26667)

MAPAVMASSATTVAPFQGLKSTAGLPISCRSGSTGLSSVSNGGRIRCMQVWPIEGIKKFE
TLSYLPPLSTEALLKQVDYLIRSKWVPCLEFSKVGFVFREHNSSPGYYDGRYWTMWKL
PMFGCTDATQVLNEVEEVKKEYPDAYVRVIGFDNMRQVQCVSFIAFRPPGCEESGKA

Barley_B hordein (uniprot: Q40026)

MKTFLIFALLVIAATSTIAQQQPFPQQPFPQQPQPYPQQPQPYPQQPFQPQQPFPQQTIPQ
QPQPYPQQPFPPQQEFPQQPPFWPQQPFPQQPPFGLQQPILSQQQPCTPQQTPLPQGQLY
QTLLQLQIPYVHPSILQQLNPCKVFLQQQCSPVRMPQLIARLQMLQQSSCHVLQQQCCQ
QLPQISEQFRHEAIRAIVYSIFLQEQPQQSVQGVSQTQQQLQQEQVGQCSFQQPQPQQLG
QAQQVPQSVFLQPHQIAQLEATTSIALRTLPRMCNVNVPLYDIMPPDFWH

Barley_D hordein (uniprot: Q40054)

MAKRLVLFVAVIVALVALTTAEREINGNNIFLDSRSRQLQCERELQESSLEACRRVVDQQ
LVGQLPWSTGLQMQCCQQLRDVSPECRPVALSQVVRQYEQQTEVPSKGGSFYPGGTAP
PLQQGGWWGTSVKWYYPDQTSSQQSWQGQQGYHQSVTSSQQPGQGQQGSYPGSTFP
QQPGQGQQPGQRQPWSYPSATFPQQPGQGQGQQGYYPGATSLLQPGQGQQGPYQSAT
SPQQPGQGQGQQETYPIATSPHQPGQWQQPGQGQQGFYPSVTSPQQSGQGQQGYPSTT
SPQQSGQGQQLGQGQQPGQGQQGYPSATFPQQPGQWQQGSYPSTTSPQQSGQGQQGY
NPSGTSTQQPGQVQQLGQGQQGYYPIATSPQQPGQGQQLGQGQQPGHGQQLVQGQQQ

GQGQQGHYPSMTSPHQTGQGQKGYYPSAISPQQSGQGQQGYQPSGASSQGSVQGACQ
HSTSSPQQQAQGCQASSPKQGLGSLYYPSGAYTQQKPGQGYNPGGTSPLHQQGGGFGG
GLTTEQPQGGKQPFHCQQTTVSPHQGQQTTVSPHQGQQTTVSPHQGQQTTVSPHQGQQ
TTVSPHQGQQTTVSPHQGQQTTVSPHPGQQTTVSPHQGQQTTVSPHQGQQTTVSPHQG
QQPGEQPCGFPGQQTTVSLHHGQQSNELYYGSPYHVSVEQPSASLKVAKAQQLAAQLP
AMCRLEGGGGLLASQ

Barley_γ-Hordein-3 (uniprot: P80198)
ITTTTMQFNPSGLELERPQQLFPQWQPLPQQPPFLQQEPEQPYPQQQPLPQQQPFPQQPQ
LPHQHQFPQQLPQQQFPQQMPLQPQQQFPQQMPLQPQQQPQFPQQKPFGQYQQPLTQQ
PYPQQQPLAQQQPSIEEQHQLNLCKEFLLQQCTLDEKVPLLQSVISFLRPHISQQNSCQL
KRQQCCQQLANINEQSRCPAIQTIVHAIVMQQQVQQQVGHGFVQSQLQQLGQGMPIQL
QQQPGQAFVLPQQQAQFKVVGSLVIQTLPMLCNVHVPPYCSPFGSMATGSGGQ

Barley_Large subunit RuBisCO (uniprot: P05698)
MSPQTETKAGVGFQAGVKDYKLTYYTPEYETKDTDILAAFRVSPQPGVPPEEAGAAVA
AESSTGTWTTVWTDGLTSLDRYKGRCYHIEPVAGEDSQWICYVAYPLDLFEEGSVTNM
FTSIVGNVFGFKALRALRLEDLRIPPTYSKTFQGPPHGIQVERDKLNKYGRPLLGCTIKP
KLGLSAKNYGRACYECLRGGLDFTKDDENVNSQPFMRWRDRFVFCAEAIYKSQAETG
EIKGHYLNATAGTCEEMIKRAVFARELGVPIVMHDYLTGGFTANTTLAHYCRDNGLLLH
IHRAMHAVIDRQKNHGMHFRVLAKALRMSGGDHIHSGTVVGKLEGEREMTLGFVDLL
RDDFIEKDRARGIFFTQDWVSMPGVIPVASGGIHVWHMPALTEIFGDDSVLQFGGGTLG
HPWGNAPGAAANRVALEACVQARNEGRDLAREGNEIIRAACKWSPELAAACEVWKAI
KFEFEPVDTIDKKV

Barley_Small subunit RuBisCO (uniprot: Q40004)
MAPTVMASSATSVAPFQGLKSTAGLPVSRRSNASSASVSNGGRIRCMQVWPIEGIKKFE
TLSYLPPLSTEALLKQVDYLIRSKWVPCLEFSKVGFIFREHNASPGYYDGRYWTMWKL
PMFGCTDATQVLNEVEEVKKEYPDAYVRIIGFDNMRQVQCVSFIAFKPPGCQESGKA

Oat_Avenin (uniprot: P27919)
MKIFFFLALLALVVSATFAQYAESDGSYEEVEGSHDRCQQHQMKLDSCREYVAERCTT
MRDFPITWPWKWWKGGCEELRNECCQLLGQMPSECRCDAIWRSIQRELGGFFGTQQG

LIGKRLKIAKSLPTQSTWALSAISPNSMVSHIAGKSSILRALPVDVLANAYRISRQEARNL
KNNRGQESGVFTPKFTQTSFQPYPEGEDESSLINKASE

Oat_11S globulin (uniprot: Q38779)
MATTSFPSMLFYFCIFLLFHGSMAQLFGQSSTPWQSSRQGGLRGCRFDRLQAFEPLRQV
RSQAGITEYFDEQNEQFRCTGVSVIRRVIEPQGLVLPQYHNAPALVYILQGRGFTGLTFPG
CPATFQQQFQPFDQSQFAQGQSQSQTIKDEHQRVQRFKQGDVVALPAGIVHWCYNDGD
APIVAIYVFDVNNNANQLEPRQKKFLLAGNNKFLLAGNNANQLEPRQKEFLLAGNNKR
EQQSGNNIFSGLSVQLLSEALGISQQAAQGSKSNDQRGRVIRVSQGLQFLKPIVSQQVPV
EQQVYQPIQTQDVQATQYQVGQSTQYQVGKSTPYQGGQSSQYQAGQSWDQSFNGLEE
NFCSLEARKNIENPQHADTYNPRAGRITRLNSKNFPILNIVQMSATRVNLYQNAILSPFW
NINAHSVIYMIQGHARVQVVNNNGQTVFSDILHRGQLLIVPQHFVVLKNAEREGCQYIS
FKTNPNSMVSHIAGKTSILRALPIDVLANAYRISRQEARNLKNNRGEEFGAFTPKLTQTG
FQSYQDIEEASSSAVRASE

Oat_12S globulin (uniprot: Q49257)
MKTMRKQIYKKAYWLLLPFLPLALANTFLVKEDSKNVTAYTPFATPITDSKSDLVSLAQ
LDSSYQIADQTIHNTNLFVLFKSRDVKVKYESSGSNNISFDSTSQGEKPSYVVEFTNSTNI
GIKWTMVKKYQLDVPNVSSDMNQVLKNLILEQPLTKYTLNSSLAKEKGKTQREVHLG
SGQANQWTSQRNQHDLNNNPSPNASTGFKLTTGNAYRKLSESWPIYEPIDGTKQGKGK
DSSGWSSTEENEAKNDAPSVSGGGSSSGTFNKYLNTKQALESIGILFDDQTPRNVITQLY
YASTSKLAVTNNHIVVMGNSFLPSMWYWVVERSAQENASNKPTWFANTNLDWGEDK
QKQFVENQLGYKETTSTNSHNFHSKSFTQPAYLISGIDSVNDQIIFSGFKAGSVGYDSSSS
SSSSSSSTKDQALAWSTTTSLDSKTGYKDLVTNDTGLNGPINGSFSIQDTFSFVVPYSGN
HTNNGTTGPIKTAYPVKKDQKSTVKINSLINATPLNSYGDEGIGVFDALGLNYNFKSNQ
ERLPSRTDQIFVYGIVSPNELRSAKSSADSTGSDTKVNWSNTQSRYLPVPYNYSEGIIDA
DGFKRPENRGASVTTFSGLKSIAPDGFANSIANFSVGLKAGIDPNPVMSGKKANYGAVV
LTRGGVVRLNFNPGNDSLLSTTDNNIAPISFSFTPFTAAESAVDLTTFKEVTYNQESGLWS
YIFDSSLKPSHDGKQTPVTDNMGFSVITVSRTGIELNQDQATTTLDVAPSALAVQSGIQST
TQTLTGVLPLSEEFSAVIAKDSDQNKIDIYKNNNGLFEIDTQLSNSVATNNGGLAPSYTEN
RVDAWGKVEFADNSVLQARNLVDKTVDEIINTPEILNSFFRFTPAFEDQKATLVATKQSD
TSLSVSPRIQFLDGNFYDLNSTIAGVPLNIGFPSRVFAGFAALPAWVIPVSVGSSVGILFILL
VLGLGIGIPMYRVRKLQDASFVNVFKKVDTLTTAVGSVYKKIITQTGVVKKAPSALKAA

NPSVKKPAAFLKPPVQPPSKPEGEQKAVEVKSEETKS

Oat_Large subunit RuBisCO (uniprot: P48684)
MSPQTETKASVGFQAGVKDYKLTYYTPEYETKDTDILAAFRVTPQPGVPPEEAGAAVA
AESSTGTWTTVWTDGLTSLDRYKGRCYHIEPVAGEDNQWICYVAYPLDLFEEGSVTNM
FTSIVGNVFGFKALRALRLEDLRIPPAYTKTFQGPPHGIQVERDKLNKYGRPLLGCTIKP
KLGLSAKNYGRACYECLRGGLDFTKDDENVNSQPFMRWRDRFVFCAEAIYKAQAETG
EIKGHYLNATAGTCEEMIKRAVFARELGVPIVMHDYITGGFTANTSLAHYCRDNGLLLH
IHRAMHAVIDRQKNHGMHFRVLAKALRMSGGDHIHSGTVVGKLEGEREMTLGFVDLL
RDDFIEKDRARGIFFTQDWVSMPGVIPVASGGIHVWHMPALTEIFGDDSVLQFGGGTLG
HPWGNAPGAAANRVALEACVQARNEGRDLAREGNEIIREACKWSPELAAACEVWKAI
KFEFEPVDTIDE

Oat_Small subunit RuBisCO (uniprot: Q9ZWG4)
VTASSATSVAPFQGLKSTAGLPVGRRSNAGLGNVSNGGRIRCMQVWPIEGIKKFETLSY
LPPLSEEALLKQIDFLIRSKWVPCLEFSKVGFIFREHGSTPGYYDGRYWTMWKLPMFGC
TDASQVIKEVEEVKKEYPDAYVRIIGFDNIRQVQCVSFIAFKPPGCE

Corn_Large subunit RuBisCO (uniprot: P00874)
MSPQTETKASVGFKAGVKDYKLTYYTPEYETKDTDILAAFRVTPQLGVPPEEAGAAVA
AESSTGTWTTVWTDGLTSLDRYKGRCYHIEPVPGDPDQYICYVAYPLDLFEEGSVTNMF
TSIVGNVFGFKALRALRLEDLRIPPAYSKTFQGPPHGIQVERDKLNKYGRPLLGCTIKPK
LGLSAKNYGRACYECLRGGLDFTKDDENVNSQPFMRWRDRFVFCAEAIYKAQAETGEI
KGHYLNATAGTCEEMIKRAVFARELGVPIVMHDYLTGGFTANTTLSHYCRDNGLLLHIH
RAMHAVIDRQKNHGMHFRVLAKALRMSGGDHIHSGTVVGKLEGEREITLGFVDLLRD
DFIEKDRSRGIFFTQDWVSMPGVIPVASGGIHVWHMPALTEIFGDDSVLQFGGGTLGHP
WGNAPGAAANRVALEACVQARNEGRDLAREGNEIIKAACKWSAELAAACEIWKEIKF
DGFKAMDTI

Corn_Small subunit RuBisCO (uniprot: P05348)
MAPTVMMASSATAVAPFQGLKSTASLPVARRSSRSLGNVSNGGRIRCMQVWPAYGNKK
FETLSYLPPLSTDDLLKQVDYLLRNGWIPCLEFSKVGFVYRENSTSPCYYDGRYWTMW
KLPMFGCNDATQVYKELQEAIKSYPDAFHRVIGFDNIKQTQCVSFIAYKPPGSD

Rice_Large subunit RuBisCO (uniprot: P0C512)

MSPQTETKASVGFKAGVKDYKLTYYTPEYETKDTDILAAFRVTPQPGVPPEEAGAAVA
AESSTGTWTTVWTDGLTSLDRYKGRCYHIEPVVGEDNQYIAYVAYPLDLFEEGSVTNM
FTSIVGNVFGFKALRALRLEDLRIPPTYSKTFQGPPHGIQVERDKLNKYGRPLLGCTIKP
KLGLSAKNYGRACYECLRGGLDFTKDDENVNSQPFMRWRDRFVFCAEAIYKSQAETG
EIKGHYLNATAGTCEEMIKRAVFARELGVPIVMHDYLTGGFTANTSLAHYCRDNGLLLH
IHRAMHAVIDRQKNHGMHFRVLAKALRMSGGDHIHAGTVVGKLEGEREMTLGFVDLL
RDDFIEKDRARGIFFTQDWVSMPGVIPVASGGIHVWHMPALTEIFGDDSVLQFGGGTLG
HPWGNAPGAAANRVALEACVQARNEGRDLAREGNEIIRSACKWSPELAAACEIWKAIK
FEFEPVDKLDS

Rice_Small subunit RuBisCO (uniprot: P18566)

MAPTVMASSATSVAPFQGLKSTAGLPVSRRSTNSGFGNVSNGGRIKCMQVWPIEGIKKF
ETLSYLPPLTVEDLLKQIEYLLRSKWVPCLEFSKVGFVYRENHRSPGYYDGRYWTMWK
LPMFGCTDATQVLKELEEAKKAYPDAFVRIIGFDNVRQVQLISFIAYKPPGCEESGGN

Sorghum_Large subunit RuBisCO (uniprot: A1E9T2)

MSPQTETKASVGFKAGVKDYKLTYYTPEYETKDTDILAAFRVTPQLGVPPEEAGAAVA
AESSTGTWTTVWTDGLTSLDRYKGRCYHIEPVPGDPDQYICYVAYPLDLFEEGSVTNMF
TSIVGNVFGFKALRALRLEDLRIPPAYVKTFQGPPHGIQVERDKLNKYGRPLLGCTIKPK
LGLSAKNYGRACYECLRGGLDFTKDDENVNSQPFMRWRDRFVFCAEAIYKAQAETGEI
KGHYLNATAGTCEEMIKRAVFAKELGVPIVMHDYLTGGFTANTTLSHYCRDNGLLLHIH
RAMHAVIDRQKNHGMHFRVLAKALRMSGGDHIHSGTVVGKLEGEREITLGFVDLLRD
DFIEKDRSRGIFFTQDWVSMPGVIPVASGGIHVWHMPALTEIFGDDSVLQFGGGTLGHP
WGNAPGAAANRVALEACVQARNEGRDLAREGNEIIKAACKWSAELAAACEIWKEIKF
DTFKAMDTL

Sorghum_Small subunit RuBisCO (uniprot: C5Y519)

MAPTVMASSATAVAPFQGLKSTATLPVARRSTTSLAKVSNGGRIRCMQVWPAYGNKKFE
TLSYLPPLTEEQLLKQVDYLLRNNWVPCLEFSKEGFVYRENSTSPCYYDGRYWTMWK
LPMFGCTDASQVYKELQEAIASYPDAYVRILGFDNIKQTQCVSFIAYKPAGSE

Soybean_Basic 7S (uniprot: P13917)

MASILHYFLALSLSCSFLFFLSDSVTPTKPINLVVLPVQNDGSTGLHWANLQKRTPLMQV
PVLVDLNGNHLWVNCEQQYSSKTYQAPFCHSTQCSRANTHQCLSCPAASRPGCHKNTC
GLMSTNPITQQTGLGELGEDVLAIHATQGSTQQLGPLVTVPQFLFSCAPSFLVQKGLPRN
TQGVAGLGHAPISLPNQLASHFGLQRQFTTCLSRYPTSKGAIIFGDAPNNMRQFQNQDIF
HDLAFTPLTITLQGEYNVRVNSIRINQHSVFPLNKISSTIVGSTSGGTMISTSTPHMVLQQS
VYQAFTQVFAQQLPKQAQVKSVAPFGLCFNSNKINAYPSVDLVMDKPNGPVWRISGED
LMVQAQPGVTCLGVMNGGMQPRAEITLGARQLEENLVVFDLARSRVGFSTSSLHSHGV
KCADLFNFANA

Soybean_Glycinin (uniprot: P04347)

MGKPFFTLSLSSLCLLLLSSACFAITSSKFNECQLNNLNALEPDHRVESEGGLIETWNSQ
HPELQCAGVTVSKRTLNRNGSHLPSYLPYPQMIIVVQGKGAIGFAFPGCPETFEKPQQQS
SRRGSRSQQQLQDSHQKIRHFNEGDVLVIPLGVPYWTYNTGDEPVVAISPLDTSNFNNQ
LDQNPRVFYLAGNPDIEHPETMQQQQQQKSHGGRKQGQHRQQEEEGGSVLSGFSKHF
LAQSFNTNEDTAEKLRSPDDERKQIVTVEGGLSVISPKWQEQEDEDEDEDEEYGRTPSY
PPRRPSHGKHEDDEDEDEEEDQPRPDHPPQRPSRPEQQEPRGRGCQTRNGVEENICTMK
LHENIARPSRADFYNPKAGRISTLNSLTLPALRQFGLSAQYVVLYRNGIYSPDWNLNANS
VTMTRGKGRVRVVNCQGNAVFDGELRRGQLLVVPQNPAVAEQGGEQGLEYVVFKTHH
NAVSSYIKDVFRVIPSEVLSNSYNLGQSQVRQLKYQGNSGPLVNP

Soybean_β-Conglycinin, β-Chain (uniprot: P25974)

MMRVRFPLLVLLGTVFLASVCVSLKVREDENNPFYFRSSNSFQTLFENQNVRIRLLQRF
NKRSPQLENLRDYRIVQFQSKPNTILLPHHADADFLLFVLSGRAILTLVNNDDRDSYNLH
PGDAQRIPAGTTYYLVNPHDHQNLKIIKLAIPVNKPGRYDDFFLSSTQAQQSYLQGFSHN
ILETSFHSEFEEINRVLFGEEEEQRQQEGVIVELSKEQIRQLSRRAKSSSRKTISSEDEPFNL
RSRNPIYSNNFGKFFEITPEKNPQLRDLDIFLSSVDINEGALLLPHFNSKAIVILVINEGDA
NIELVGIKEQQQKQKQEEEPLEVQRYRAELSEDDVFVIPAAYPFVVNATSNLNFLAFGIN
AENNQRNFLAGEKDNVVRQIERQVQELAFPGSAQDVERLLKKQRESYFVDAQPQQKE
EGSKGRKGPFPSILGALY

Soybean_β-Conglycinin, α-Chain (uniprot: P13916)

MMRARFPLLLLGLVFLASVSVSFGIAYWEKENPKHNKCLQSCNSERDSYRNQACHARC

NLLKVEKEECEEGEIPRPRPRPQHPEREPQQPGEKEEDEDEQPRPIPFPRPQPRQEEEHEQ
REEQEWPRKEEKRGEKGSEEEDEDEDEEQDERQFPFPRPPHQKEERNEEEDEDEEQQRE
SEESEDSELRRHKNKNPFLFGSNRFETLFKNQYGRIRVLQRFNQRSPQLQNLRDYRILEF
NSKPNTLLLPNHADADYLIVILNGTAILSLVNNDDRDSYRLQSGDALRVPSGTTYYVVN
PDNNENLRLITLAIPVNKPGRFESFFLSSTEAQQSYLQGFSRNILEASYDTKFEEINKVLFS
REEGQQQGEQRLQESVIVEISKEQIRALSKRAKSSSRKTISSEDKPFNLRSRDPIYSNKLG
KFFEITPEKNPQLRDLDIFLSIVDMNEGALLLPHFNSKAIVILVINEGDANIELVGLKEQQ
QEQQQEEQPLEVRKYRAELSEQDIFVIPAGYPVVVNATSNLNFFAIGINAENNQRNFLAG
SQDNVISQIPSQVQELAFPGSAQAVEKLLKNQRESYFVDAQPKKKEEGNKGRKGPLSSI
LRAFY

Soybean_β-Conglycinin, α’-Chain (uniprot: P11827)
MMRARFPLLLLGVVFLASVSVSFGIAYWEKQNPSHNKCLRSCNSEKDSYRNQACHARC
NLLKVEEEEECEEGQIPRPRPQHPERERQQHGEKEEDEGEQPRPFPFPRPRQPHQEEEHE
QKEEHEWHRKEEKHGGKGSEEEQDEREHPRPHQPHQKEEEKHEWQHKQEKHQGKES
EEEEEDQDEDEEQDKESQESEGSESQREPRRHKNKNPFHFNSKRFQTLFKNQYGHVRV
LQRFNKRSQQLQNLRDYRILEFNSKPNTLLLPHHADADYLIVILNGTAILTLVNNDDRDS
YNLQSGDALRVPAGTTFYVVNPDNDENLRMIAGTTFYVVNPDNDENLRMITLAIPVNK
PGRFESFFLSSTQAQQSYLQGFSKNILEASYDTKFEEINKVLFGREEGQQQGEERLQESVI
VEISKKQIRELSKHAKSSSRKTISSEDKPFNLGSRDPIYSNKLGKLFEITQRNPQLRDLDV
FLSVVDMNEGALFLPHFNSKAIVVLVINEGEANIELVGIKEQQQRQQQEEQPLEVRKYR
AELSEQDIFVIPAGYPVMVNATSDLNFFAFGINAENNQRNFLAGSKDNVISQIPSQVQEL
AFPRSAKDIENLIKSQSESYFVDAQPQQKEEGNKGRKGPLSSILRAFY

Quinoa_RuBisCO large chain (uniprot: K4P448)
PQPGVPPEEAGAAVAAESSTGTWTTVWTDGLTSLDRYKGRCYHIEPVAGEENQYICYVA
YPLDLFEEGSVTNMFTSIVGNVFGFKALRALRLEDLRIPVAYIKTFQGPPHGIQVERDKL
NKYGRPLLGCTIKPKLGLSAKNYGRACYECLRGGLDFTKDDENVNSQPFMRWRDRFLF
CAEAIYKAQAETGEIKGHYLNATAATCEEMIKRAVFARELGAPIVMHDYLTGGFTANTS
LAHYCRDNGLLLHIHRAMHAVIDRQKNHGMHFRVLAKALRLSGGDHIHSGTVVGKLE
GERDITLGFVDLLRDDYTEKDRSRGIYFTQSWVSTPGVLPVASGGIHVWHMPALTEIFG
DDSVLQFGGGTLGHPWGNAPGAVANRVALEACVQARNEGRDL

Canola_Cruciferin (uniprot: P11090)

MARLSSLLSFSLALLTFLHGSTAQQFPNECQLDQLNALEPSHVLKAEAGRIEVWDHHAP
QLRCSGVSFVRYIIESKGLYLPSFFSTARLSFVAKGEGLMGRVVLCAETFQDSSVFQPSGG
SPFGEGQGQGQQGQGQGHQGQGQGQQGQQGQQGQQSQGQGFRDMHQKVEHIRTGD
TIATHPGVAQWFYNDGNQPLVIVSVLDLASHQNQLDRNPRPFYLAGNNPQGQVWIEGR
EQQPQKNILNGFTPEVLAKAFKIDVRTAQQLQNQQDNRGNIIRVQGPFSVIRPPLRSQRP
QEEVNGLEETICSARCTDNLDDPSNADVYKPQLGYISTLNSYDLPILRFLRLSALRGSIR
QNAMVLPQWNANANAVLYVTDGEAHVQVVNDNGDRVFDGQVSQGQLLSIPQGFSVV
KRATSEQFRWIEFKTNANAQINTLAGRTSVLRGLPLEVISNGYQISLEEARRVKFNTIETT
LTHSSGPASYGGPRKADA

Canola_Napin small chain & Napin large chain (uniprot: P17333)

MANKLFLVSATLAFFFLLTNASIYRTIVEVDEDDATNPAGPFRIPKCRKEFQQAQHLKAC
QQWLHKQAMQSGSGPSWTLDGEFDFEDDMENPQGPQQRPPLLQQCCNELHQEEPLCV
CPTLKGASKAVKQQVRQQQGQQGQQLQQVISRIYQTATHLPKVCNIPQVSVCPFQKTM
PGPSY

Amaranth_Large subunit RuBisCO (uniprot: P16306)

MSPQTETKASVGFKAGVKDYRLTYYTPEYETQDTDILAAFRVSPQPGVPPEEAGAAVAA
ESSTGTWTSVWTDGLTNLDRYKGRCYNIEPVAGEENQYICYVAYPLDLFEEGSVTNMFT
SIVGNVFGFKALRALRLEDLRIPVAYVKTFQGPPHGIQVERDKLNKYGRPLLGCTIKPKL
GLSAKNYGRACYECLRGGLDFTKDDENVNSQPFMRWRDRFLFCAEAIYKSQAETGEIK
GHYLNATAGTCEEMIKRAVFARELGVPIVMHDYLTGGFTANTSLSQYCRDNGLLLHIHV
AMHAVIDRQKNHGMHFRVLAKALRLSGGDHIHSGTVVGKLEGERDITLGFVDLLRDD
YTEKDRCRGIFFTQSWVSTPGVLPVASGGIHVWHMPALTEIFGDDSVLQFGGGTLGHPW
GNAPGAVANRVALEACVQARNEGRDLAREGNTIIREAAKWSPELAAACEVWKEIKFEF
PAMDTI

Palamaria palmata_50S ribosomal protein L31,chloroplastic (uniprot: Q5MIM5)

MPQIDIHPEWYNDSKVYCDGKHIMTIGSTKPELHVDIWSGNHPFFTGSQRIIDTEGRVER
FMRKYNVQKTADT

Palamaria palmata_PALPL Ribosomal protein 12 (uniprot: Q5MIL8)

MPTIQQLVRLPRQKAVKKTKSPALKACPQRRGVCTRVYTTTPKKPNSALRKVQESITSG

Palamaria palmata_Allophycocyanin α chain (uniprot: M1UZ22)
MSIVTKSIVNADAEARYLSPGELDRIKSFVLSGQRRLRIAQILTENRERIVKQGGQQLFQ
KRPDVVSPGGNAYGEEMTATCLRDLDYYLRLVSYGIVAGDVTPIEEIGLVGVKEMYNSL
GTPISGVAEGVRSMKNVACSLLSGEDSAEAGFYFDYTLGAMQ

Palamaria palmata_Allophycocyanin β chain (uniprot: M1VJV1)
MQDAITSVINAADVQGKYLDDSSVEKLRGYFQTGELRVRAAATIAANAATIIKESVAKS
LLYSDITRPGGNMYTTRRYAACIRDLDYYLRYATYGMLAGDPSILDERVLNGLKETYNS
LGVPIGATIQAIQAMKEVTIGLVGPDAGKEMGLYFDYICSGLS

Palamaria palmata_Phycocyanin α (uniprot: I2FJU0)
MKTPITEAIASADSQGRFLSNAELQSINGRYERASSSLEAAASLTNSAQRLITGAAQAVY
TKFPFTTQMPGPTYASSAIGKAKCARDIGYYLRMTTYCLVVGATGPMDEYLVAGLEEIN
RSFELSPSWYIEALQYIKSSHGLSGQVGNEANTYVDYAINTLS

Palamaria palmata_Phycocyanin β (uniprot: I2FJT9)
MLDAFAKVVAQADARGEFLSNTQLDALSTMVNEGKKRLDVVNKINANASAIVTNSAR
ALFAEQPQLVQPGGNAYTSRRMAACLRDMEIVLRYVSYSMVAGDSSVLDDRCLNGLRE
TYQALGTPGTSVAVAIQKMKEASVALANDLNNVPLGDCSALTAELGSYFDRAAIAVV

Palamaria palmata_Phycoerythrin α subunit (uniprot: F2ZAL8)
MKSVMTTTISAADAAGRFPSSSDLESVQGNIQRAAARLEAAEKLASNHEAVVKEGGDA
CFAKYSYLKNPGEAGDSQEKVNKCYRDVDHYMRLVNYSLVVGGTGPLDEWAIAGARE
VYRTLNLPSASYVAAFAFTRDRLCVPRDMSAQAGGEYVAALDYIVNALT

Palamaria palmata_Phycoerythrin β subunit (uniprot: F2ZAL7)
MLDAFSRVVVNSDAKAAYVGGSDLQALKKFITDGNKRLDSVSFVVSNASCIVSDAVSG
MICENPGLIAPGGNCYTNRRMAACLRDGEIILRYASYALLAGDPSVLEDRCLNGLKETYI
ALGVPTNSSVRAVSIMKASATAFVSGTASDRKMACPDGDCSALASELGSYCDRVAAAIS

Palamaria palmata_RuBisCO large chain (uniprot: Q9THF8)

MSQSVEQRTRIKNERYESGVIPYAKMGYWDPEHVILETDVLALFRVTPQPGVDPIEASA
AIAGESSTATWTVVWTDLLTACDLYRAKCYKVDAVPNTSDQYFAYIAYDIDLFEEGSIAN
LTASIIGNVFGFKAVKALRLEDMRLPVAYLKTFQGPATGTVVERERMDKFGRPFLGATV
KPKLGLSGKNYGRVVYEGLKGGLDFLKDDENINSQPFMRYSERYLYSMEGVNRAQAA
AGEIKGHYLNVTAATMENMYERAEFAQQLGSVICMIDLVIGYSAIQSMAIWARKTDMIL
HLHRAGNSTYSRQKIHGMNFRVICKWMRMAGVDHIHAGTVVGKLEGDPVMIQGFYN
TLLESHLEANLPQGIFFEQDWASLRKCTPVASGGIHCGQMHQLLDYLGDDVVLQFGGG
TIGHPDGIQAGATANRVALESMVIARNEGRDFVAEGPQILRDAAKTCGPLQTALDLWKD
ISFNYTSTDTADFVETPTANV

Palamaria palmata_RuBisCO small chain (uniprot: O98734)
MRLTQGAFSFLPDLTDDQISKQVSYAMGKGWSVNIEYTDDPHPRNSYWELWGLPLFDIP
DAPTVMYEISSCRKAHPGVYIKVNAFDNTRGVESCSLSFIINRPASEPGFGLLRTEDIGRN
QKYAIHSYATEKPEGSRY

Palamaria palmata_Small subunit RuBisCO (uniprot: Q9XGX5)
MASSMMSNAATAVAVAATSGGAQANMVAPFNGLKSIASFPVTRKSNDITSIASNGGRVQ
CMQVWPPVGKKKFETLSYLPPLSDAQLLAQVQYLLNKGWIPCIEFELEHPFVYRENHR
SPGYQDGRYWTMWKLPMYGCTDPAQVLNEVEEAKKAYPTAFIRIIGFDNKRQVQCVSF
IAYKPADSY

Chicken egg_Ovalbumin (uniprot: P01012)
MGSIGAASMEFCFDVFKELKVHHANENIFYCPIAIMSALAMVYLGAKDSTRTQINKVV
RFDKLPGFGDSIEAQCGTSVNVHSSLRDILNQITKPNDVYSFSLASRLYAEERYPILPEYL
QCVKELYRGGLEPINFQTAADQARELINSWVESQTNGIIRNVLQPSSVDSQTAMVLVNAI
VFKGLWEKAFKDEDTQAMPFRVTEQESKPVQMMYQIGLFRVASMASEKMKILELPFAS
GTMSMLVLLPDEVSGLEQLESIINFEKLTEWTSSNVMEERKIKVYLPRMKMEEKYNLTS
VLMAMGITDVFSSSANLSGISSAESLKISQAVHAAHAEINEAGREVVGSAEAGVDAASV
SEEFRADHPFLFCIKHIATNAVLFFGRCVSP

Chicken egg_Ovotransferrin (uniprot: P02789)
MKLILCTVLSLGIAAVCFAAPPKSVIRWCTISSPEEKKCNNLRDLTQQERISLTCVQKATY
LDCIKAIANNEADAISLDGGQAFEAGLAPYKLKPIAAEVYEHTEGSTTSYYAVAVVKKG

TEFTVNDLQGKTSCHTGLGRSAGWNIPIGTLLHRGAIEWEGIESGSVEQAVAKFFSASCV
PGATIEQKLCRQCKGDPKTKCARNAPYSGYSGAFHCLKDGKGDVAFVKHTTVNENAP
DQKDEYELLCLDGSRQPVDNYKTCNWARVAAHAVVARDDNKVEDIWSFLSKAQSDFG
VDTKSDFHLFGPPGKKDPVLKDLLFKDSAIMLKRVPSLMDSQLYLGFEYYSAIQSMRKD
QLTPSPRENRIQWCAVGKDEKSKCDRWSVVSNGDVECTVVDETKDCIIKIMKGEADAV
ALDGGLVYTAGVCGLVPVMAERYDDESQCSKTDERPASYFAVAVARKDSNVNWNNLK
GKKSCHTAVGRTAGWVIPMGLIHNRTGTCNFDEYFSEGCAPGSPPNSRLCQLCQGSGGI
PPEKCVASSHEKYFGYTGALRCLVEKGDVAFIQHSTVEENTGGKNKADWAKNLQMDD
FELLCTDGRRANVMDYRECNLAEVPTHAVVVRPEKANKIRDLLERQEKRFGVNGSEKS
KFMMFESQNKDLLFKDLTKCLFKVREGTTYKEFLGDKFYTVISSLKTCNPSDILQMCSF
LEGK

Chicken egg_Ovomucoid (uniprot: P01005)
MAMAGVFVLFSFVLCGFLPDAAFGAEVDCSRFPNATDKEGKDVLVCNKDLRPICGTDG
VTYTNDCLLCAYSIEFGTNISKEHDGECKETVPMNCSSYANTTSEDGKVMVLCNRAFNP
VCGTDGVTYDNECLLCAHKVEQGASVDKRHDGGCRKELAAVSVDCSEYPKPDCTAED
RPLCGSDNKTYGNKCNFCNAVVESNGTLTLSHFGKC

Bovine milk_αs1-Casein (uniprot: P02662)
MKLLILTCLVAVALARPKHPIKHQGLPQEVLNENLLRFFVAPFPEVFGKEKVNELSKDIGS
ESTEDQAMEDIKQMEAESISSSEEIVPNSVEQKHIQKEDVPSERYLGYLEQLLRLKKYKV
PQLEIVPNSAEERLHSMKEGIHAQQKEPMIGVNQELAYFYPELFRQFYQLDAYPSGAWY
YVPLGTQYTDAPSFSDIPNPIGSENSEKTTMPLW

Bovine milk_αs2-Casein (uniprot: P02663)
MKFFIFTCLLAVALAKNTMEHVSSSEESIISQETYKQEKNMAINPSKENLCSTFCKEVVR
NANEEEYSIGSSSEESAEVATEEVKITVDDKHYQKALNEINQFYQKFPQYLQYLYQGPIV
LNPWDQVKRNAVPITPTLNREQLSTSEENSKKTVDMESTEVFTKKTKLTEEEKNRLNFL
KKISQRYQKFALPQYLKTVYQHQKAMKPWIQPKTKVIPYVRYL

Bovine milk_β-Casein (uniprot: P02666)
MKVLILACLVALALARELEELNVPGEIVESLSSSEESITRINKKIEKFQSEEQQQTEDELQ
DKIHPFAQTQSLVYPFPGPIPNSLPQNIPPLTQTPVVVPPFLQPEVMGVSKVKEAMAPKH

KEMPFPKYPVEPFTESQSLTLTDVENLHLPLPLLQSWMHQPHQPLPPTVMFPPQSVLSLS
QSKVLPVPQKAVPYPQRDMPIQAFLLYQEPVLGPVRGPFPIIV

Bovine milk_κ-Casein (uniprot: P02668)
MMKSFFLVVTILALTLPFLGAQEQNQEQPIRCEKDERFFSDKIAKYIPIQYVLSRYPSYGL
NYYQQKPVALINNQFLPYPYYAKPAAVRSPAQILQWQVLSNTVPAKSCQAQPTTMARH
PHPHLSFMAIPPKKNQDKTEIPTINTIASGEPTSTPTTEAVESTVATLEDSPEVIESPPEINT
VQVTSTAV

Bovine milk_β-Lactoglobulin (uniprot: P02754)
MKCLLLALALTCGAQALIVTQTMKGLDIQKVAGTWYSLAMAASDISLLDAQSAPLRVY
VEELKPTPEGDLEILLQKWENGECAQKKIIAEKTKIPAVFKIDALNENKVLVLDTDYKKY
LLFCMENSAEPEQSLACQCLVRTPEVDDEALEKFDKALKALPMHIRLSFNPTQLEEQCH
I

Bovine milk_α-Lactalbumin (uniprot: P00711)
MMSFVSLLLVGILFHATQAEQLTKCEVFRELKDLKGYGGVSLPEWVCTTFHTSGYDTQ
AIVQNNDSTEYGLFQINNKIWCKDDQNPHSSNICNISCDKFLDDDLTDDIMCVKKILDK
VGINYWLAHKALCSEKLDQWLCEKL

Bovine milk_Lactoferrin (uniprot: P24627)
MKLFVPALLSLGALGLCLAAPRKNVRWCTISQPEWFKCRRWQWRMKKLGAPSITCVR
RAFALECIRAIAEKKADAVTLDGGMVFEAGRDPYKLRPVAAEIYGTKESPQTHYYAVAV
VKKGSNFQLDQLQGRKSCHTGLGRSAGWIIPMGILRPYLSWTESLEPLQGAVAKFFSAS
CVPCIDRQAYPNLCQLCKGEGENQCACSSREPYFGYSGAFKCLQDGAGDVAFVKETTV
FENLPEKADRDQYELLCLNNSRAPVDAFKECHLAQVPSHAVVARSVDGKEDLIWKLLS
KAQEKFGKNKSRSFQLFGSPPGQRDLLFKDSALGFLRIPSKVDSALYLGSRYLTTLKNLR
ETAEEVKARYTRVVWCAVGPEEQKKCQQWSQQSGQNVTCATASTTDDCIVLVLKGEA
DALNLDGGYIYTAGKCGLVPVLAENRKSSKHSSLDCVLRPTEGYLAVAVVKKANEGLT
WNSLKDKKSCHTAVDRTAGWNIPMGLIVNQTGSCAFDEFFSQSCAPGADPKSRLCALC
AGDDQGLDKCVPNSKEKYYGYTGAFRCLAEDVGDVAFVKNDTVWENTNGESTADWA
KNLNREDFRLLCLDGTRKPVTEAQSCHLAVAPNHAVVSRSDRAAHVKQVLLHQQALFG
KNGKNCPDKFCLFKSETKNLLFNDNTECLAKLGGRPTYEEYLGTEYVTAIANLKKCSTS

PLLEACAFLTR

Bovine milk_BSA (uniprot: P02769)
MKWVTFISLLLLFSSAYSRGVFRRDTHKSEIAHRFKDLGEEHFKGLVLIAFSQYLQQCPF
DEHVKLVNELTEFAKTCVADESHAGCEKSLHTLFGDELCKVASLRETYGDMADCCEKQ
EPERNECFLSHKDDSPDLPKLKPDPNTLCDEFKADEKKFWGKYLYEIARRHPYFYAPEL
LYYANKYNGVFQECCQAEDKGACLLPKIETMREKVLASSARQRLRCASIQKFGERALK
AWSVARLSQKFPKAEFVEVTKLVTDLTKVHKECCHGDLLECADDRADLAKYICDNQDT
ISSKLKECCDKPLLEKSHCIAEVEKDAIPENLPPLTADFAEDKDVCKNYQEAKDAFLGSF
LYEYSRRHPEYAVSVLLRLAKEYEATLEECCAKDDPHACYSTVFDKLKHLVDEPQNLIK
QNCDQFEKLGEYGFQNALIVRYTRKVPQVSTPTLVEVSRSLGKVGTRCCTKPESERMPC
TEDYLSLILNRLCVLHEKTPVSEKVTKCCTESLVNRRPCFSALTPDETYVPKAFDEKLFT
FHADICTLPDTEKQIKKQTALVELLKHKPKATEEQLKTVMENFVAFVDKCCAADDKEA
CFAVEGPKLVVSTQTALA

Bovine meat_Myosin-1 (uniprot: Q9BE40)
MSSDQEMAVFGEAAPYLRKSEKERIEAQNKPFDAKTSVFVADPKESFVKATVQSREGG
KVTAKTEAGATVTVKEDQVFPMNPPKFDKIEDMAMMTHLHEPAVLYNLKERYAAWMI
YTYSGLFCVTVNPYKWLPVYNAEVVTAYRGKKRQEAPPHIFSISDNAYQFMLTDRENQ
SILITGESGAGKTVNTKRVIQYFATIAVTGEKKKEEPTSGKMQGTLEDQIISANPLLEAFG
NAKTVRNDNSSRFGKFIRIHFGTTGKLASADIETYLLEKSRVTFQLKAERSYHIFYQIMS
NKKPELIEMLLITTNPYDYAYVSQGEITVPSIDDQEELMATDSAIEILGFTSDERVSIYKLT
GAVMHYGNLKFKQKQREEQAEPDGTEVADKAAYLQGLNSADLLKALCYPRVKVGNEF
VTKGQTVEQVYNAVGALAKAVYDKMFLWMVARINQQLDTKQPRQYFIGVLDIAGFEIF
DFNSLEQLCINFTNEKLQQFFNHHMFVLEQEEYKKEGIEWEFIDFGMDLAACIELIEKPM
GIFSILEEECMFPKATDMSFKNKLYEQHLGKSNNFQKPKPAKGKAEAHFSLIHYAGTVD
YNITGWLDKNKDPLNETVVGLYQKSSVKTLALLFSGPASGEAEGGPKKGGKKKGSSFQ
TVSALFRENLNKLMTNLRSTHPHFVRCIIPNETKTPGAMEHELVLHQLRCNGVLEGIRIC
RKGFPSRILYADFKQRYKVLNASAIPEGQFIDSKKASEKLLASIDVDHTQYKFGHTKVFF
KAGLLGLLEEMRDEKLAQLITRTQARCRGFLARVEYQKMVERRESIFCIQYNVRAFMN
VKHWPWMKLYFKIKPLLKSAETEKEMANMKEEFEKTKEELAKSEAKRKELEEKMVTL
TQEKNDLQLQVQSEADALADAEERCDQLIKTKIQLEAKIKEVTERAEDEEEINAELTAK
KRKLEDECSELKKDIDDLELTLAKVEKEKHATENKVKNLTEEMAGLDETIAKLTKEKK

ALQEAHQQTLDDLQAEEDKVNTLTKAKTKLEQQVDDLEGSLEQEKKLRMDLERAKRK
LEGDLKLAQESTMDIENDKQQLDEKLKKKEFEMSNLQSKIEDEQALAMQLQKKIKELQ
ARIEELEEEIEAERASRAKAEKQRSDLSRELEEISERLEEAGGATSAQIEMNKKREAEFQ
KMRRDLEEATLQHEATAAALRKKHADSVAELGEQIDNLQRVKQKLEKEKSEMKMEID
DLASNMETVSKAKGNLEKMCRALEDQLSELKTKEDEQQRLINDLTTQRARLQTESGEF
SRQLDEKDALVSQLSRGKQAFTQQIEELKRQLEEEIKAKSALAHALQSARHDCDLLREQ
YEEEQEGKAELQRAMSKANSEVAQWRTKYETDAIQRTEELEEAKKKLAQRLQDAEEH
VEAVNAKCASLEKTKQRLQNEVEDLMIDVERTNAACAALDKKQRNFDKILSEWKQKY
EETHAELEASQKESRSLSTELFKIKNAYEESLDQLETLKRENKNLQQEISDLTEQIAEGG
KRIHELEKVKKQVEQEKSEIQAALEEAEASLEHEEGKILRIQLELNQVKSEIDRKIAEKD
EEIDQLKRNHIRIVESMQSTLDAEIRSRNDAIRLKKKMEGDLNEMEIQLNHANRMAAEA
LKNYRSTQAILKDTQIHLDDALRGQEDLKEQLAMVERRANLLQAEIEELRATLEQTERS
RKIAEQELLDASERVQLLHTQNTSLINTKKKLETDITQIQGEMEDIIQEARNAEEKAKKA
ITDAAMMAEELKKEQDTSAHLERMKKNLEQTVKDLQHRLDEAEQLALKGGKKQIQKL
EARVRELEGEVESEQKRNVEAVKGLRKHERRVKELTYQTEEDRKNILRLQDLVDKLQA
KVKSYKRQAEEAEEQSNVNLSKFRKLQHELEEAEERADIAESQVNKLRVKSREVHTKII
SEE

Bovine meat_Myosin regulatory light chain 12B (uniprot: A4IF97)
MSSKKAKTKTKKRPQRATSNVFAMFDQSQIQEFKEAFNMIDQNRDGFIDKEDLHDMLA
SLGKNPTDAYLEAMMNEAPGPINFTMFLTMFGEKLNGTDPEDVIRNAFACFDEEATGTI
QEDYLRELLTTMGDRFTDEEVDELYREAPIDKKGNFNYIEFTRILKHGAKDKDD

Bovine meat_Actin, cytoplasmin 1 (uniprot: P60712)
MDDDIAALVVDNGSGMCKAGFAGDDAPRAVFPSIVGRPRHQGVMVGMGQKDSYVGD
EAQSKRGILTLKYPIEHGIVTNWDDMEKIWHHTFYNELRVAPEEHPVLLTEAPLNPKAN
REKMTQIMFETFNTPAMYVAIQAVLSLYASGRTTGIVMDSGDGVTHTVPIYEGYALPHAI
LRLDLAGRDLTDYLMKILTERGYSFTTTAEREIVRDIKEKLCYVALDFEQEMATAASSSS
LEKSYELPDGQVITIGNERFRCPEALFQPSFLGMESCGIHETTFNSIMKCDVDIRKDLYAN
TVLSGGTTMYPGIADRMQKEITALAPSTMKIKIIAPPERKYSVWIGGSILASLSTFQQMW
ISKQEYDESGPSIVHRKCF

Bovine meat_Collagen α-1 (III) chain (uniprot: P02459)

MIRLGAPQTLVLLTLLVAAVLRCHGQDVQKAGSCVQDGQRYNDKDVWKPEPCRICVC
DTGTVLCDDIICEDMKDCLSPETPFGECCPICSADLPTASGQPGPKGQKGEPGDIKDIVG
PKGPPGPQGPAGEQGPRGDRGDKGEKGAPGPRGRDGEPGTPGNPGPPGPPGPPGPPGLG
GNFAAQMAGGFDEKAGGAQMGVMQGPMGPMGPRGPPGPAGAPGPQGFQGNPGEPGE
PGVSGPMGPRGPPGPPGKPGDDGEAGKPGKSGERGPPGPQGARGFPGTPGLPGVKGHR
GYPGLDGAKGEAGAPGVKGESGSPGENGSPGPMGPRGLPGERGRTGPAGAAGARGND
GQPGPAGPPGPVGPAGGPGFPGAPGAKGEAGPTGARGPEGAQGPRGEPGTPGSPGPAG
AAGNPGTDGIPGAKGSAGAPGIAGAPGFPGPRGPPGPQGATGPLGPKGQTGEPGIAGFK
GEQGPKGEPGPAGPQGAPGPAGEEGKRGARGEPGGAGPAGPPGERGAPGNRGFPGQDG
LAGPKGAPGERGPSGLAGPKGANGDPGRPGEPGLPGARGLTGRPGDAGPQGKVGPSG
APGEDGRPGPPGPQGARGQPGVMGFPGPKGANGEPGKAGEKGLPGAPGLRGLPGKDG
ETGAAGPPGPAGPAGERGEQGAPGPSGFQGLPGPPGPPGEGGKPGDQGVPGEAGAPGL
VGPRGERGFPGERGSPGSQGLQGARGLPGTPGTDGPKGAAGPAGPPGAQGPPGLQGMP
GERGAAGIAGPKGDRGDVGEKGPEGAPGKDGGRGLTGPIGPPGPAGANGEKGEVGPPG
PAGTAGARGAPGERGETGPPGPAGFAGPPGADGQPGAKGEQGEAGQKGDAGAPGPQG
PSGAPGPQGPTGVTGPKGARGAQGPPGATGFPGAAGRVGPPGSNGNPGPPGPPGPSGK
DGPKGARGDSGPPGRAGDPGLQGPAGPPGEKGEPGDDGPSGPDGPPGPQGLAGQRGIV
GLPGQRGERGFPGLPGPSGEPGKQGAPGASGDRGPPGPVGPPGLTGPAGEPGREGSPGA
DGPPGRDGAAGVKGDRGETGAVGAPGAPGPPGSPGPAGPIGKQGDRGEAGAQGPMGP
AGPAGARGMPGPQGPRGDKGETGEAGERGLKGHRGFTGLQGLPGPPGPSGDQGASGP
AGPSGPRGPPGPVGPSGKDGANGIPGPIGPPGPRGRSGETGPAGPPGNPGPPGPPGPPGP
GIDMSAFAGLGQREKGPDPLQYMRADEAAGNLRQHDAEVDATLKSLNNQIESLRSPEG
SRKNPARTCRDLKLCHPEWKSGDYWIDPNQGCTLDAMKVFCNMETGETCVYPNPASV
PKKNWWSSKSKDKKHIWFGETINGGFHFSYGDDNLAPNTANVQMTFLRLLSTEGSQNI
TYHCKNSIAYLDEAAGNLKKALLIQGSNDVEIRAEGNSRFTYTVLKDGCTKHTGKWGK
TMIEYRSQKTSRLPIIDIAPMDIGGPEQEFGVDIGPVCFL

Pig_β-Actin (uniprot: Q8SPK6)
TPAMYVAIQAVLSLYASGRTTGIVMDSGDGVTHTVPIYEGYALPHAILRLDLAGRDLTDY
LMKILTERGYSFTTTAEREIVRDIKEKLCYVALDFEQEMATAASSSSLEKSYELPDGQVIT
IGNERFRCPEALFQPSFLGMESCGIHETTFNSIMKCDVDIRKDLYANT

Pig_Actin, α skeletal muscle (uniprot: P68137)

MCDEDETTALVCDNGSGLVKAGFAGDDAPRAVFPSIVGRPRHQGVMVGMGQKDSYVG
DEAQSKRGILTLKYPIEHGIITNWDDMEKIWHHTFYNELRVAPEEHPTLLTEAPLNPKAN
REKMTQIMFETFNVPAMYVAIQAVLSLYASGRTTGIVLDSGDGVTHNVPIYEGYALPHAI
MRLDLAGRDLTDYLMKILTERGYSFVTTAEREIVRDIKEKLCYVALDFENEMATAASSSS
LEKSYELPDGQVITIGNERFRCPETLFQPSFIGMESAGIHETTYNSIMKCDIDIRKDLYAN
NVMSGGTTMYPGIADRMQKEITALAPSTMKIKIIAPPERKYSVWIGGSILASLSTFQQM
WITKQEYDEAGPSIVHRKCF

Pig_Myosin light chain (uniprot: Q29069)
MSFSADQIAEFKEAFLLFDRTGECKITLSQVGDVLRALGTNPTNAEVKKVLGNPSNEEM
NAKKIEFEQFLPMLQAISNNKDQGSYEDFVEGLRVFDKEGNGTVMVTELRHVLATLGE
KMKEEEVEALMAGQEDSNGCINYEAFVKHIMSI

Pig_Myosin heavy chain (uniprot: Q29623)
MSSDQEMAIFGEAAPYLRKSEKERIEAQNRPFDAKTSVFVAEPKESFVKGTIQSREGGK
VTVKTEAGATLTVKEDQVFPMNPPKFDKIEDMAMMTHLHEPGVLYNLKERYAAWMIY
TYSGLFCVTVNPYKWLPVYNPEVVTAYRGKKRQEA

Atlantic salmon_Actin, cytoplasmic 1 (uniprot: O42161)
MEDEIAALVVDNGSGMCKAGFAGDDAPRAVFPSIVGRPRHQGVMVGMGQKDSYVGD
EAQSKRGILTLKYPIEHGIVTNWDDMEKIWHHTFYNELRVAPEEHPVLLTEAPLNPKAN
REKMTQIMFETFNTPAMYVAIQAVLSLYASGRTTGIVMDSGDGVTHTVPIYEGYALPHAI
LRLDLAGRDLTDYLMKILTERGYSFTTTAEREIVRDIKEKLCYVALDFEQEMGTAASSSS
LEKSYELPDGQVITIGNERFRCPEALFQPSFLGMESCGIHETTYNSIMKCDVDIRKDLYA
NTVLSGGTTMYPGIADRMQKEITSLAPSTMKIKIIAPPERKYSVWIGGSILASLSTFQQM
WISKQEYDESGPSIVHRKCF

Atlantic salmon_Myosin regulatory light chain 2 (uniprot: Q7ZZN0)
MAPKKAKRRGAAAEGGSSNVFSMFEQSQIQEYKEAFTIIDQNRDGIISKDDLRDVLASM
GQLNVKNEELEAMVKEASGPINFTVFLTMFGEKLKGADPEDVIVSAFKVLDPEATGFIK
KEFLQELLTTQCDRFSAEEMKNLWAAFPPDVAGNVDYKQICYVITHGEEKEE

Atlantic salmon_Slow myosin heavy chain (uniprot: Q2HXU3)

MGDTLMAEFGGAASYLRKSDKERMECQTRPFDIKRECYVPDPEVEYVKATITSRDGAK
VTVDTEFGKTVTVKEDDIHPQNPPKFDKIEDMAMFTFLHEPAVLFNLKERYAAWMIYTY
SGLFCVTVNPYKWLPVYDQSVVNAYRGKKRTEAPPHIFSISDNAYQYMLSDRENQSVLI
TGESGAGKTVNTKRVIQYFASIAAVGGKKSAAEEKKGTLEDQIIQANPALEA

Atlantic salmon_Collagen Type XI α2 (uniprot: A7KE05)
MDIRDCPFRKKRRPWRVDLSSFALVTLVVALCQLPVARADPVDVLRMLQLPSLPEGVRK
VPGFCTSRRAGTADHAYRISKKAQISAPTNQLFSGRFPENFSIMALVKAHAGLQAFLLSI
YSEQGVQQLGVELGRSPVFLYEDQTGKPAPEDYPLFSGVNLADGKWHRIAISVSKKNV
TLLLDCKKRMTRALPRSKSPVVDTKGITVFGARLLDEEVFQGDIQQLLIASNPQAAYDF
CEHYSPDCDSPLPKTQSQDPNTHVEGTLVAEEVELAKAEGEAEAFTEEYVTGDLGMKE
YDYSYKDYNEPLPEGESDGYMGPALSAVTDEGGGMLIEGPPGPEGQAGLPGPNGPSGPP
GSVGDPGERGLPGKAGIAGADGVPGPPGTSVMLPFRFGSSGGDKGPGVSAQEAQAQAI
LSQARLALKGPPGPMGYTGRTGPLGTPGSPGLKGEGGDPGPQGPRGPQGLSGPPGKSG
RRGRAGADGARGMPGEGGPKGDRGFDGLPGLPGDKGHRGDTGGLGPQGGPGEDGER
GDDGDIGPRGLPGEPGPRGLLGPKGPNGIPGPPGVRGNDGPHGPKGNLGPQGEPGPPGQ
QGTSGTQGMPGPQGAHGPPGEKGPTGKPGLPGMSGADGPPGHPGKEGPVGTKGNQGP
NGPQGTIGYPGPRGIKGTQGIRGLKGHKGEKGEDGFPGIKGDFGVKGERGEGGVPGPR
GEDGPEGPKGRVGPPGELGPIGLVGEKGSLGFPGFPGTNGEKGTRGTSGSDGPSGPPGER
GLPGPQGANGFPGPKGPPGPPGKDGLPGHPGQRGEVGPSGETGPLGERGHPGPPGPPGE
QGLSGPSGKEGTKGDPGPPGGPGKDGPPGLRGFPGERGMPGTPGEKGPIGPAGRDGVQ
GPVGLPGAAGSIGVPGEDGDKASNHRDISITYSNCCVYHVKRYHNAHFKHLLTKAFLV
DFWDYRGADGELGARGQQGPFGAKGDEGSRGFPGAPGPIGLQGPQGPPGGVGNPGNL
GDKGEPGEAGPPGIGGEPGKKGPVGFPGDPGPPGELGPRGSPGPTGENGPPGGPGKRGL
AGTRGPEGRQGEKGTKGDPGANGPPGKTGPVGPQGQPGKPGTEGLRGLPGSVGPPGLP
GLRGEGGDKGEKGHSGLIGLIGPTGEQGEKGDRGLPGPQGTHGSKGETFADDTTVVGLI
TDNDETAYREEVRDLAVWCQDNKLSLNVSKTKEMIVDYRKRRAEQAPIYIDGAVVERI
LKKFYSCTIESNLTGCITAWYGNCSASDCNALQGGVSGGTGPIGPGGPAGMPGPEGVKG
AKGATGGAGPKGEKGVSGPPGPPGPPGEVIQPMPIQISKKSKRSIDASQMLSETDAESAA
ADATGPEFLTGREGMEEIFGSLNSLRQEIETMRFPLGTKGSPARTCQDLKLSQPELKDGE
YWIDPNQGCSRDSLKVFCNFTAGGETCLYPSKTVDTVKLNSWTTEKPGSWYSEFATGS
KLSYVDSNGEPIGVVQLGFLRLLSVQARQNFTYHCHRSVAWADRTANNGHKRALHFQG
ANDEDLSYETSPYIKALVDGCSYRKGFDSTVVEVNTPQVEQLPLRDLKITDFGENNQKF

GFEVGPVCFQG

Chum salmon_Type 1 collagen α2 chain (uniprot: Q8UUJ4)

MLSFVDNRILLLLAVTSLLASCQSGPRGAKGPRGDRGPQGPNGRDGKAGLPGVAGPPG
PPGLGGNFAAQFDGGKGSDPGPGPMGLMGSRGPNGPPGSPGPQGFTGHAGEPGEPGQT
GSIGARGPTGSAGKPGEDGNNGRPGKPGDRGGPGTQGARGFPGTPGLPGMKGHRGYN
GLDGRKGESGTAGAKGETGAHGANGTPGPAGSRGLNGERGRAGPAGPAGARGADGST
GPAGPAGPLGAAGPPGFPGAPGPKGEIGGAGSNGPSGPQGGRGEPGINGAVGPVGPVGN
PGNNGINGAKGAAGLPGVAGAPGFPGPRGGPGPQGPQGSTGARGLGGDPGPSGQKGDS
GAKGEPGHSGVQGAAGPAGEEGKRGSTGEAGATGPAGLRGARGGAGTRGLPGLEGRG
GPIGMPGARGATGPAGIRGAPGDAGRAGESGLTGARGLPGNSGQGGPPGKEGPSGAAG
LDGRTGPPGPTGPRGQPGNIGFPGPKGPGGEAGKGGDKGPTGATGLRGGPGADGNNGA
PGPAGVVGNAGEKGEQGPSGAPGFQGLPGPAGPAGEAGKAGNQGMPGDQGLPGPAGV
KGERGNSGPAGSAGSQGAIGARGPAGTPGPDGGKGEPGSVGIVGAAGHQGPGGMPGE
RGAGGTPGPKGEKGEGGHRGLEGNMGRDGARGAAGPSGPPGPSGANGEKGESGSFGP
AGPAGLRGPSGERGEGGPAGPPGFAGPPGSDGQSGPRGEKGPAGGKGDVGPAGPAGPS
GQSGPSGASGPAGPPGGRGDAGPSGLTGFPGAAGRVGGPGPAGISGPPGSAGPAGKDGP
RGLRGDAGPGGPQGEQGVVGPAGIAGDKGPSGEGGPPGAPGTAGPQGVLGPSGFVGLP
GSRGDKGLPGGPGAVGEPGRLGPAGASGPRGPSGNIGMPGMTGTQGEAGREGNSGND
GPPGRPGAAGFKGDRGEPGSPGALGSSGQPGPNGPAGSAGRPGNRGESGPTGNGGPVG
AAGARGAPGPAGPRGEKGGAGEKGDRGMKGLRGHGGLQGMPGPNGPSGETGSAGIT
GPAGPRGPAGPHGPPGKDGRAGGHGAIGPVGHRGPPGHLGPAGPPGSPGLPGPAGPAGG
GYDQSGGYDEYRADQPSLRAKDYEVDATIKSLNSQIENLLTPEGSKKNPARTCRDIRLS
HPEWSSGFYWIGPNQGCIADAIKAYCDFSTGHTCIHPHPESIARKNWYRSSENKKHVWF
GETINGGTEFAYNDETLSPQSMATQLAFMRLLANQATQNITYHCKNSVAYMDGENGNL
KKAVLLQGSNDVELRAEGNSRFTFNVLEDGCTRHTGQWSKTVIEYRTNKPSRLPILDIA
PLDIGEADQEFGLDIGPVCFK

Chum salmon_Actin (uniprot: Q9PVN1)

MCDDDETTALVCDNGSGLVKAGFAGDDAPRAVFPSIVGRPRHQGVMVGMGQKDSYV
GDEAQSKRGILTLKYPIEHGIITNWDDMEKIWHHTFYNELRVAPEEHPTLLTEAPLNPKA
NREKMTQIMFETFNVPAMYVAIQAVLSLYASGRTTGIVLDSGDGVTHNVPIYEGYALPH
AIMRLDLAGRDLTDYLMKILTERGYSFVTTAEREIVRDIKEKLCYVALDFENEMATAASS

SSLEKSYELPDGQVITIGNERFRCPETLFQPSFIGMESAGIHETAYNSIMKCDIDIRKDLYA
NNVLSGGTTMYPGIADRMQKEITALAPSTMKIKIIAPPERKYSVWIGGSILASLSTFQAM
WITKQEYDEAGPSIVHRKCF

Chum salmon_β-Actin (uniprot: J7ID80)
EHGIVTNWDDMEKIWHHTFYNELRVAPEEHPVLLTEAPLNPKANREKMTQIMFETFNT
PAMYVAIQAVLSLYASGRTTGIVMDSGDGVTHTVPIYEGYALPHAILRLDLAGRDLTDYL
MKILTERGYSFTTTAEREIVRDIKEKLCYVALDFEQEMGTAASSSSLEKSYELPDGQVITI
GNERFR

Chum salmon_Myosin heavy chain (uniprot: Q8JIP5)
MSTDAEMQAYGKAAIYLRKSEKERMEAQATPFDSKNACYVTDKVELYLKGLVTARAD
GKCTVTVTNPDGSKEEGKEFEEADIYEMNPPKYDKIEDMAMMTYLNEASVLYNLKER
YAAWMIYTYSGLFCATVNPYKWLPVYDEEVVNAYRGKKRMEAPPHIFSVSDNAFQFM
MIDKENQSILITGESGAGKTVNTKCVIQYFATIAVSGSKKEVDPSKMQGSLEDQIIAANPL
LESYGNAKTVRNDNSSRFGKFIRIHFQAGKLAKADIETYLLEKSRVAFQLPDERGYHIFY
QLMTGHKPELVEMTLLTTNPYDFPMISQGHIAVPSINDKEELDATDDAITILGFTNDEKM
SIYKLTGAVTHHGNLKFKQKQREEQAEPDGTEVADKIGYLLGLNSAELLKCLCYPRVKV
GNEYVTKGQTVAQVYNAVMALAKSIYERMFLWMVIRINEMLDTKNPRQFYIGVLDIAG
FEIFDYNSMEQLCINFTNEKLQQFFNHTMFVLEQEEYKKEGIVWEFIDFGMDLAACIELI
EKPLGIFSILEEECMFPKASDTTFKNKFYDQHLGKTKAFEKPKPAKGKPEAHFSLVHYAG
TVDYNITGWLDKNKDPLNESVILMYGKASVKLLATLYPAAPPEDKAKKGGKKKGGSM
QTVSSQFRENLHKLMTNLRSTHPHFVRCLIPNESKTPGLMENFLVIHQLRCNGVLEDLRI
CRKGFPSRIIYADFKQRYKVLNASVIPEGQFMDNKKASEKLLGSIDVNHEDYKFGHTKV
FFKAGLLGVLEEMRDEKLAALVGMVQALSRGFLMRREFSKMMERRESIFSIQYNIRSF
MNVKTWPWMKLYFKIKPLLQSAETEKELANMKENYEKMKTDLAKALATKKHLEEKL
VALVQERADLALQVASEGQSLNDAEERCEGLIKSKIQLEAKLKEMTERLEDEEEMNAEL
TAKKRKLEDECSELKKDIDDLELTLAKVEKEKHATENKVKNLTEEMASLDESVAKLTKE
KKALQEAHQQTLDDLQAEEDKVNTLTKARTKLEQQVDDLEGSLEQEKKLRMDLERAK
RKLEGDLKLAQESIMDLENDKQQADEKIKKKEFETSQLLSKVEDEQSLGAQLQKKIKEL
QARIEELEEEIEAERAARAKVERQRADLSRELEEISERLEEAGGATSAQIDMNKKREAEF
QKLRRDLEESTLQHEATAAALRKKQADSVAELGEQIDNLQRVKQKLEKEKSEYKMEID
DLSSNMEAVAKAKGNLEKMCRTLEDQLSELKTKNDENVRQVNDISGQRARLLTENGEF

GRQLEEKEALVSQLTRGKQAFTQQVEELKRQIEEEVKAKNALAHGVQSARHDCDLLRE
QFEEEQEAKAELQRGMSKANSEVAQWRTKYETDAIQRTEELEEAKKKLAQRLQDAEET
IEATNSKCSSLEKTKQRLQGEVEDLMIDVERANAMAANLDKKQRNFDKVLAEWKQKY
EEGQAELEGAQKEARSMSTELFKLKNSYEEALDHLETLKRENKNLQQEISDLTEQIGET
GKSIHELEKAKKTVETEKSEIQTALEEAEGTLEHEESKILRVQLELNQIKGEVDRKIAEK
DEEMEQIKRNSQRVVDSMQSTLDSEVRSRNDALRVKKKMEGDLNEMEIQLSHSNRQAS
EAQKQLRNVQGQLKDAQLHLDDAVRVAEDMKEQAAMVERRNGLMVAEIEELRVALE
QTERGRKVAETELVDASERVGLLHSQNTSLLNTKKKLETDLVQVQGEVDDIIQEARNAE
EKAKKAITDAAMMAEELKKEQDTSSHLERMKKNLEVTVKDLQHRLDEAENLAMKGG
KKQLQKLEWRVRELETEVEAEQRRGVDAVKGVRKYERRVKELTYQTEEDKKNVGRLQ
DLVDKLQMKVKAYKRHAEEAEEAANQHMSKFRKVQHELEEAEERADIAETQVNKLR
AKTRDSGKGKEVAE

Tuna_β-Actin (uniprot: A9CM08)
PIYEGDARPHAIRRLDLAGRDLTDYLMKIRTERGYSFTTTGEREIVRDIKEKLCYVALDF
EQEMGTAASSSSLEKSYELPDGQVITIGNERFRCPEALFQPSFLGMESCGIHETTFNSIMK
CDVDIRKDLYANTVLSGGTTMYPGIADRMQKEITAL

Tuna_Myosin heavy chain-1 (uniprot: G9M5T1)
MSTDAEMEAYGPAAIYLRKPEKERIEAQTAPFDAKTAFFVTDKEEMYLKGKLVKREGG
KATVETDCGKTLTVKEDEIFPRNPPNFDKIEDMAMMTHLNEPCVLYNLKERFASWMIYT
YSGLFCVVVNPYKWLPVYDAVVVGGYRGKKRIEAPPHIFSISDNAYQFMHTDRENQSIL
ITGESGAGKTVNTKRVIQYFATIAAIGAKKAEPTPGKMQGSLEDQIVAANPLLESYGNAK
TVRNDNSSRFGKFIRIHFGSTGKLASADIETYLLEKSRVTFQLSAERSYHIFYQLMTGHQ
PELLEGLLITTNPYDYPMVSQGEITVKSIDDVEEFIATDTAIDILGFTAEEKMGIYKLTGAV
MHHGNMKFKQKQREEQAEPDGTEVADKISYLLGLNSADMLKYLCYPRVKVGNEMVT
KGQTVPQVNNAVSALCKSVYDREFLWMVIRINEMLDTKQPRQYFIGVLDIAGFEIFDFN
SLEQLCINFTNEKLQQFFNHHMFVLEQEEYKKEGIVWEFIDFGMDLAACIELIEKPMGIF
SILEEECMFPKASDTTFKNKLHDQHLGKTKAFEKPKPVKGKPEAHFSLVHYAGTVDYNI
TGWLDKNKDPLNDSVVQLYQKASNKLLAFLYAKHGAADEGGGGKKGKKKGGSFQTV
SALFRENLGKLMTNLRSTHPHFVRCLIPNESKTPGLMENFLVIHQLRCNGVLEGIRICRK
GFPSRILYGDFKQRYKVLNASVIPEGQFIDNKKAAEKLLGSIDVDHTQYKFGHTKVFFK
AGLLGLLEEMRDEKLANLVPMTQALCRGFLMRLEFVKMMERREAVFSIQYNIRSFMNV

KNWPWMNLYFKIKPLLKSAETEKELMNMKENYEKMKTDLATALAKKKELEEKMVSL
LQEKNDLQLEVASETENLSDAEERCEGLIKSKIQLEAKLKETTERLEDEEEINAELTAKK
RKLEDECSELKKDIDDLELTLAKVEKEKHATENKVKNLTEEMASQDESIAKLTKEKKAL
QEAHQQTLDDLQAEEDKVNTLTKAKTKLEQQVDDLEGSLEQEKKLRMDLERAKRKLE
GDLKLAQESIMDLENDKQQSDEKIKKKEFETSQLLSKIEDEQSLGAQLQKKIKELQARIE
ELEEEIEAERAARAKVEKQRADLSRELEEISERLEEAGGATAAQIEMNKKREAEFQKLR
RDLEESTLQHEATSASLRKKQADSVAELGEQIDNLQRVKQKLEKEKSEYKMEIDDLSSN
MEAVAKSKGNLEKMCRTIEDQLSELKAKNDEHVRQLNDLNGQRARLQTENGEFSRQIE
EKDALVSQLTRGKQAYTQQIEELKRHIEEEIKAKNALAHAVQSARHDCDLLREQYEEEQ
EAKGELQRGMSKANSEVAQWRTKYETDAIQRTEELEEAKKKLAQRLQDAEESIEAVNS
KCASLEKTKQRLQGEVEDLMIDVERANSLAANLDKKQRNFDKVLADWKQKYEEGQS
ELEGAQKEARSLSTELFKMKNSYEEALDHLETMKRENKNLQQEISDLTEQIGETGKSIH
ELEKAKKHVETEKTEIQTALEEAEGTLEHEEAKILRVQLELNQIKSEVDRKLAEKDEEM
EQIKRNSQRVIDSMQSTLDAEVRSRNDALRIKKKMEGDLNEMEIQLSHANRQATESQK
QLRNVQGQLKDAQLHLDDAVRGHEDMKEQVAMVERRNGLMLAEIEELRAALEQTER
GRKVAEQELVDASERVGLLHSQNTSLINTKKKLEADLVHIQGEVDDSIQEARNAEDKAK
KAITDAAMMAEELKKEQDTSAHLERMKKNLEVSVKDLQHRLDEAEALAMKGGKKQL
QKLESRVRELESEVDAESRRGADAIKGVRKYERRVKELTYQTEEDKKNVHRLQDLVDK
LQLKVKSYKRQAEEAEEQANTHLSRYRKVQHEMEEAQERADIAESQVNKLRAKSRDH
HHGKGEHAE

Tuna_Myosin heavy chain-2 (uniprot: G9M5T2)
MSTDAEMECYGPAAIYLRKPERERIEAQTAPFDAKTAFFVTEKEEMYLKGKLVNREGG
KATVDTDCGKTVTVKEDEIFPRNPPNFDKIEDMAMMTHLNEPCVLYNLKERFASWMIY
TYSGLFCVVVNPYKWLPVYDSQVVVAYRGKKRIEAPPHIFSISDNAYQFMHTDRENQSI
LITGESGAGKTVNTKRVIQYFATIAAIGAKKAEPTPGKMQGSLEDQIVAANPLLESYGNA
KTVRNDNSSRFGKFIRIHFGSTGKLASADIETYLLEKSRVTFQLSAERSYHIFYQLMTGH
QPELLEGLLITTNPYDYPMVSQGEITVKSIDDVEEFIATDTAIDILGFTAEEKMGIYKLTG
AVMHHGNMKFKQKQREEQAEPDGTEVADKISYLLGLNSADMLKYLCYPRVKVGNEM
VTKGQTVPQVNNAVSALCKSVYDREFLWMVIRINEMLDTKQPRQFFIGVLDIAGFEIFD
FNSLEQLCINFTNEKLQQFFNHHMFVLEQEEYKKEGIVWEFIDFGMDLAACIELIEKPM
GIFSILEEECMFPKASDTTFKNKLHDQHLGKTKAFEKPKPVKGKPEAHFSLVHYAGTVD
YNITGWLDKNKDPLNDSVVQLYQKASNKLLAFLYAKHGAADEGGGGKKGKKKGGSF

QTVSALFRENLGKLMTNLRSTHPHFVRCLIPNESKTPGLMENFLVIHQLRCNGVLEGIRI
CRKGFPSRILYGDFKQRYKVLNASVIPEGQFIDNKKAAEKLLGSIDVDHTQYKFGHTKV
FFKAGLLGLLEEMRDEKLALLVPMTQALCRGFLMRTEFVKMMERREAIFSIQYNIRSFM
NVKNWPWMNLYFKIKPLLKSAETEKELMNMKENYEKMKTDLATALAKKKELEEKMV
SLLQEKNDLQLEVASETENLSDAEERCEGLIKSKIQLEAKLKETTERLEDEEEINAELTAK
KRKLEDECSELKKDIDDLELTLAKVEKEKHATENKVKNLTEEMASQDESIAKLTKEKKA
LQEAHQQTLDDLQAEEDKVNTLTKAKTKLEQQVDDLEGSLEQEKKLRMDLERAKRKL
EGDLKLAQESIMDLENDKQQSDEKIKKKEFETSQLLSKIEDEQSLGAQLQKKIKELQARI
EELEEEIEAERAARAKVEKQRADLSRELEEISERLEEAGGATAAQIEMNKKREAEFQKL
RRDLEESTLQHEATSASLRKKQADSVAELGEQIDNLQRVKQKLEKEKSEYKMEIDDLSS
NMEAVAKSKGNLEKMCRTIEDQLSELKAKNDEHVRQLNDLNGQRARLQTENGEFSRQI
EEKDALVSQLTRGKQAYTQQIEELKRHIEEEIKAKNALAHAVQSARHDCDLLREQYEEE
QEAKGELQRGMSKANSEVAQWRTKYETDAIQRTEELEEAKKKLAQRLQDAEESIEAVN
SKCASLEKTKQRLQGEVEDLMIDVERANSLAANLDKKQRNFDKVLADWKQKYEEGQ
SELEGAQKEARSLSTELFKMKNSYEEALDHLETMKRENKNLQQEISDLTEQIGETGKSI
HELEKAKKHVETEKTEIQTALEEAEGTLEHEEAKILRVQLELNQIKSEVDRKLAEKDEE
MEQIKRNSQRVIDSMQSTLDAEVRSRNDALRIKKKMEGDLNEMEIQLSHANRQATESQ
KQLRNVQGQLKDAQLHLDDAVRGHEDMKEQVAMVERRNGLMLAEIEELRAALEQTE
RGRKVAEQELVDASERVGLLHSQNTSLINTKKKLEADLVHIQGEVDDSIQEARNAEDKA
KKAITDAAMMAEELKKEQDTSAHLERMKKNLEVSVKDLQHRLDEAEALAMKGGKKQ
LQKLESRVRELESEVDAESRRGADAIKGVRKYERRVKELTYQTEEDKKNVHRLQDLVD
KLQLKVKSYKRQAEEAEEQANTHLSRYRKVQHEMEEAQERADIAESQVNKLRAKSRD
HHHGKGEHAE
