# Supplementary material for: Comprehensive Epitope Analysis of Monoclonal Antibodies Binding to Hen Egg Ovalbumin Using a Peptide Array
Source: Foods. 2024 Jan 26;13(3):407. doi: 10.3390/foods13030407 (PMC10855139; doi:10.3390/foods13030407)
Supplement: Supplementary file 1 [file foods-13-00407-s001.zip › Supplementary information 2.pdf]

Supplementary information 2. Model food preparation methods. Following the method, five types of model processed foods (udon noodles, pork ham, processed chicken breast, lactic fermenting beverage, and pumpkin soup) were prepared.

Udon noodles

Flour, salt, and water were combined in a mixer to form udon dough. Hen egg white solution was then incorporated to achieve ovalbumin concentrations of 0, 1, 5 μg/g in the final product. The dough was vacuum-sealed and heated in a 75°C water bath for 30 min to produce Udon noodles. These noodles were subsequently homogenized in a food processor to prepare the samples.

Pork ham

Pork, salt, phosphates, sodium nitrite, and sodium ascorbate were blended in a food processor. For targeted ovalbumin concentrations (0, 1, 5 μg/g) in the final product, hen egg white solution was added and mixed. The mixture was vacuum-sealed and then heat-treated in a 75°C water bath for 30 min. The resulting Pork ham was homogenized for sample preparation.

Processed chicken breast

Chicken breast, sugar, salt, and lemon juice were processed in a food processor. To attain the desired ovalbumin concentrations of 0, 1, 5 μg/g in the end product, hen egg white solution was blended in. After vacuum-sealing, the mixture was heated in an 80°C water bath for 30 min. The Processed chicken breast was then homogenized for sampling.

Lactic fermenting beverage

A commercially available lactic fermenting beverage (Yakult 400 by Yakult Honsha Co., Ltd., consisting of glucose-fructose syrup, sugar, skim milk powder, and flavoring) was used. Hen egg white solution was added to achieve ovalbumin concentrations of 0, 1, 5 μg/g. The blend was homogenized with a juicer mixer to create the beverage sample.

Pumpkin soup

Pumpkin, onion, milk, butter, salt, and water were pureed using a juicer mixer. Hen egg white solution was then added to achieve ovalbumin levels of 0, 1, 5 μg/g. After homogenization, the mixture was heated in boiling water (approximately 100°C) for 10 min, finalizing the pumpkin soup sample.
